# Size matters: how sample size affects the reproducibility and specificity of gene set analysis

**DOI:** 10.1186/s40246-019-0226-2

**Published:** 2019-10-22

**Authors:** Farhad Maleki, Katie Ovens, Ian McQuillan, Anthony J. Kusalik

**Affiliations:** Department of Computer Science, University of Saskatchewan, 110 Science Place, Saskatoon, Canada

**Keywords:** Gene expression, Gene set analysis, Enrichment analysis, Sample size, Specificity

## Abstract

**Background:**

Gene set analysis is a well-established approach for interpretation of data from high-throughput gene expression studies. Achieving reproducible results is an essential requirement in such studies. One factor of a gene expression experiment that can affect reproducibility is the choice of sample size. However, choosing an appropriate sample size can be difficult, especially because the choice may be method-dependent. Further, sample size choice can have unexpected effects on specificity.

**Results:**

In this paper, we report on a systematic, quantitative approach to study the effect of sample size on the reproducibility of the results from 13 gene set analysis methods. We also investigate the impact of sample size on the specificity of these methods. Rather than relying on synthetic data, the proposed approach uses real expression datasets to offer an accurate and reliable evaluation.

**Conclusion:**

Our findings show that, as a general pattern, the results of gene set analysis become more reproducible as sample size increases. However, the extent of reproducibility and the rate at which it increases vary from method to method. In addition, even in the absence of differential expression, some gene set analysis methods report a large number of false positives, and increasing sample size does not lead to reducing these false positives. The results of this research can be used when selecting a gene set analysis method from those available.

**Electronic supplementary material:**

The online version of this article (10.1186/s40246-019-0226-2) contains supplementary material, which is available to authorized users.

## Introduction

The choice of sample size is an important decision to make when designing a gene expression experiment. Choosing an appropriate sample size for obtaining a desired statistical power is feasible for basic statistical procedures; however, making such choices for complex procedures such as gene set analysis is not straightforward. To the best of our knowledge, there is no methodological approach to determine the optimal sample size for reaching a predetermined statistical power or achieving reproducible results in gene set analysis, where sample size refers to the number of biological replicates per treatment, tissue, or condition. Consequently, researchers either use the largest possible number of samples considering available resources—such as funding, specimens, and technicians—for conducting the experiments, or they use an arbitrary sample size—as small as two or three samples per treatment. Using unnecessarily large sample sizes wastes resources and might involve ethical concerns. On the other hand, using small sample sizes may yield unreliable and irreproducible results.

The impact of the number of used samples on the results of differential expression analyses has been studied. Tsai et al. [1] suggested a methodology for sample size estimation. They assumed an equal standardized effect size and a constant gene-gene correlation for differentially expressed genes. Relying on these assumptions, they estimated an appropriate sample size as that which led to the highest number of true positives using a beta-binomial distribution for the two-sample z-test. Their proposed approach, as they reported, might underestimate the number of required samples when the gene-gene correlation is not constant. Stretch et al. [2] reported that using a small number of samples may lead to irreproducible results in differential expression studies. Schurch et al. [3] evaluated 11 tools for differential expression analysis using a dataset with 48 controls and 48 cases. The results of these methods when using subsets of 3 controls and 3 cases were compared to the results when using all samples. They reported that for 8 methods only 20 to 40% of the differentially expressed genes were among the genes reported when using all samples. Furthermore, they suggested that to increase this percentage to a value larger than 85%, at least 20 samples per treatment are required.

Gene expression analysis typically reports several hundred genes as differentially expressed. Biological interpretation of such a large number of genes is laborious and prone to investigator bias(es) in favour of, or against the hypothesis under study. The main aim of gene set analysis—also known as enrichment analysis—is to alleviate these problems. Many gene set analysis methods are available. These methods, unlike basic statistical tests, are complex procedures; therefore, estimating sample size for obtaining a predetermined statistical power or reproducible results is challenging.

In this paper, we extend an earlier work on sample size and reproducibility in the context of gene set analysis [4]. We study a comprehensive list of 13 gene set analysis methods: PAGE [5], GAGE [6], Camera [7], ROAST [8], FRY (from the R package *limma*) [9], GSEA (both gene permutation (GSEA-G) and sample permutation (GSEA-S) versions) [10], ssGSEA [11], GSVA [12], PLAGE [13], GlobalTest [14], PADOG [15], and over-representation analysis (ORA) [16]. All of the methods can be used for pairwise comparison of phenotypes or treatments (e.g. case versus control). Using real datasets, we evaluate the reproducibility of the results of these methods across sample sizes that are commonly used in gene expression studies.

In addition, we assess the specificity of gene set analysis methods across sample sizes. Tarca et al. [17] evaluated the specificity of gene set analysis methods by calculating the number of false positives for datasets generated from permutations of sample/phenotype labels of actual expression datasets. Since after permutation of sample labels there should be no association between differential enrichment of gene sets and phenotypes, all gene sets predicted as differentially enriched by a method were considered as false positives. However, each group of samples corresponding to a condition (case or control) in their generated datasets contained a mixture of both case and control samples from the original dataset; therefore, the characteristics of the generated dataset could be different from that of the original dataset. Furthermore, they did not evaluate the specificity of gene set analysis methods across sample sizes. To avoid the shortcoming of the approach used by Tarca et al., we conduct an experiment by generating datasets of various sizes, where case and control samples for each generated dataset are the result of sampling without replacement from control samples of an actual expression dataset. Since in the generated datasets control and case samples are from actual controls, any gene set predicted as differentially enriched by a method can be considered a false positive.

## Methods

### Data

Repositories such as Gene Expression Omnibus (GEO) [18] and ArrayExpress [19] make large scale expression datasets publically available. In this research, three such case-control datasets with unrelated phenotypes from the *Affymetrix GeneChip Human Genome U133 Plus 2.0* microarray platform were obtained from GEO: 1) renal cell carcinoma tissue (77 controls and 77 cases, GSE53757) [20], 2) gingival tissues (64 controls and 183 cases, GSE10334) [21], and 3) skin tissue in psoriasis patients (64 controls and 58 cases, GSE13355) [22]. The raw data were preprocessed with the *GEOquery v2.46.15* R package and normalized with *justRMA* normalization from the *affy v1.56.0* package.

MDS (multidimensional scaling) plots in Additional file 1: Figures S1, S2, and S3 visualize the similarity between samples, respectively, for datasets GSE53757, GSE10334, and GSE13355. These plots illustrate that dataset GSE10334 has the lowest intra-class similarity and less distinction between control and case samples, while dataset GSE13355 has highest intra-class similarity and shows a clear distinction between case and control samples. All visualizations in this paper are produced for the dataset with intermediate characteristics, GSE53757, unless otherwise noted.

Probe IDs were converted to their corresponding Entrez gene identifiers using the *hgu133plus2.db v3.2.3* R package. To avoid over-emphasizing genes with a large number of probes on the arrays, it is a common practice in gene set analysis to collapse duplicate IDs. This was accomplished by using the *collapseRows* function from *WGCNA v1.61* with the *MaxMean* method that selects the probe that has the maximum average value across samples when multiple probes map to the same gene. Collapsing the probes resulted in 20,514 genes in each experiment from an initial 54,675 probes.

### Methodology

A proper study of the effect of sample size on the results of gene set analysis methods requires conducting expression studies with datasets of various sizes. These studies must be conducted while all potentially confounding factors such as phenotype under study, the platform for measuring gene expression, experiment protocol, laboratory technician skill level, and environmental conditions stay constant. Datasets of various sizes for which these factors are constant is not currently available. In this research, we utilize a systematic, quantitative approach to study the effect of sample size on the reproducibility and specificity of gene set analysis methods using large scale publically available gene expression datasets.

Assume that *D* is a dataset containing *n*_*C*_ control samples and *n*_*T*_ case samples, where both *n*_*C*_ and *n*_*T*_ are relatively large numbers (>50). Given an integer *n*, where *n*<*n*_*C*_, and *n*<*n*_*T*_, we generate a balanced case-control dataset by randomly selecting *n* samples from the *n*_*C*_ controls of *D* and *n* samples from the *n*_*T*_ case samples of *D*. The random sampling is performed without replacement; therefore, the chosen samples are unique within the generated dataset. Hereafter, we refer to such a balanced case-control dataset as a replicate dataset of size 2×*n* and the entire process for assembling a replicate dataset as the data generation procedure. Also, to avoid confusion, we refer to *D* as the original dataset.

To obtain results that do not depend on a specific composition of samples, for each given *n*, we repeat the dataset generation procedure *m* times to construct *m* replicate datasets of size 2×*n*. These replicate datasets are then used for downstream analysis. Due to the nature of random sampling, these replicate datasets are different.

The dataset generation procedure assembles replicate datasets from an original dataset; therefore, confounding factors remain almost invariable. For instance, all these replicate datasets have the same platform and use the same experiment protocol; they also have been made by the same technician(s). This makes it possible to study the effect of sample size on the result of gene set analysis methods while keeping the confounding factors nearly constant.

Different gene set analysis methods then are applied to replicate datasets of various sample sizes and their results are used to investigate the effect of sample size on the reproducibility and specificity of gene set analysis.

In this research, 13 widely used gene set analysis methods are studied—PAGE [5], GAGE [6], Camera [7], ROAST [8], FRY [9], GSEA [10], ssGSEA [11], GSVA [12], PLAGE [13], PADOG [15], GlobalTest [14], and ORA [16].

The following R packages are used for conducting gene set analysis. PLAGE, GSVA, and ssGSEA are obtained from *GSVA* package version *1.18.0*; the *phyper* method from the *stats* package version *3.4.4* is used to implement ORA; the *GSEA.1.0.R* script downloaded from the Broad Institute software page for GSEA are used to run GSEA-S and GSEA-G; ROAST, FRY, and Camera are run using the *limma* package version *3.34.9*; GAGE and PAGE are obtained from the *gage* package version *2.20.1*. For each sample size *n* (*n*∈{3,…,20}), *m*=10 replicate datasets of size 2×*n* are generated. Then a gene set analysis method is applied to each replicate dataset. For all gene set analysis methods the default parameters are used. A Benjamini-Hochberg correction [23] with a false discovery rate of 0.05 is performed for a fair comparison across methods. Also, the GO gene sets are extracted from *MSigDB* version *6.1* [10] and used as the gene set database for all experiments. Hereafter, this database is referred to as $\mathbb {G}$.

After generating $D_{1}^{(2\times n)}, \dots, D_{m}^{(2\times n)}$, each replicate dataset $D_{i}^{(2\times n)} (1\leq i \leq m)$ and the gene set database $\mathbb {G}$ are used as inputs to a gene set analysis method *ψ*, and the results after correction for multiple comparisons are stored in a vector $R_{D_{i}^{(2\times n)}}^{\psi }$. The *k*^*th*^ component of this vector is the adjusted *p*-value resulting from testing the differential enrichment of the *k*^*th*^ gene set of $\mathbb {G}$; therefore, the length of $R_{D_{i}^{(2\times n)}}^{\psi }$ is equal to the number of gene sets in $\mathbb {G}$.

The differential enrichment status of the *k*^*th*^ gene set in $\mathbb {G}$ is determined by comparing the *k*^*th*^ component of $R_{D_{i}^{(2\times n)}}^{\psi }$ against a significance level *α*=0.05. For each element of $R_{D_{i}^{(2\times n)}}^{\psi }$ that is less than *α*, the corresponding gene set in $\mathbb {G}$ is considered as differentially enriched, and non-differentially enriched otherwise. We denote the set of all gene sets predicted as differentially enriched by $S_{D_{i}^{(2\times n)}}^{\psi }$.

We use the Jaccard similarity coefficient, also known as Jaccard index [24], to quantify the reproducibility of the results of a gene set analysis method *ψ* when applied to replicate datasets $D_{i}^{(2\times n)}$ and $D_{j}^{(2\times n)}$. The Jaccard similarity coefficient is defined as follows:

1 $$  J\left(S_{D_{i}^{(2\times n)}}, S_{D_{j}^{(2\times n)}}\right) = \frac{S_{D_{i}^{(2\times n)}} \cap S_{D_{j}^{(2\times n)}}}{S_{D_{i}^{(2\times n)}} \cup S_{D_{j}^{(2\times n)}}}  $$

A Jaccard similarity coefficient of 0 indicates no overlap, i.e. no agreement, between $S_{D_{i}^{(2\times n)}}^{\psi }$ and $S_{D_{j}^{(2\times n)}}^{\psi }$, and a value of 1 indicates complete overlap, i.e. $S_{D_{i}^{(2\times n)}}^{\psi } = S_{D_{j}^{(2\times n)}}^{\psi }$. Hereafter, we refer to the Jaccard similarity coefficient as the overlap score.

For each pair of replicate datasets $D_{i}^{(2\times n)}$ and $D_{j}^{(2\times n)}$ (1≤*i*,*j*≤*m*), we calculate $J\left (S_{D_{i}^{(2\times n)}}, S_{D_{j}^{(2\times n)}}\right)$, the overlap between the sets of gene sets predicted as differentially enriched by method *ψ* when analysing $D_{i}^{(2\times n)}$ and $D_{j}^{(2\times n)}$. The resulting overlap score is stored in position (*i*,*j*) of an upper triangular matrix, which is called an overlap matrix and visualized in the “Results” section. Since $J\left (S_{D_{i}^{(2\times n)}}, S_{D_{j}^{(2\times n)}}\right) = J\left (S_{D_{j}^{(2\times n)}}, S_{D_{i}^{(2\times n)}}\right)$, for each sample size 2×*n* we need to calculate $\frac {m\times (m-1)}{2}$ overlap scores. Overlap scores $J\left (S_{D_{i}^{(2\times n)}}, S_{D_{j}^{(2\times n)}}\right)$ (1≤*i*,*j*≤*m*) indicate the extent to which the results of a gene set analysis method is reproducible when analyzing replicate datasets of size 2×*n*. High overlap indicates that method *ψ* using datasets of size 2×*n* yield reproducible results. For each method *ψ*, we conduct a Kruskal-Wallis test to statistically assess if there is a significant difference between the overlap scores across sample sizes (3≤*n*≤20). The overlap scores for each sample size 2×*n* is represented as a multiset $P_{(2 \times n)}^{\psi }$, which is a set but with repetition allowed. $P_{(2 \times n)}^{\psi }$ is defined as follows:

2 $$  P_{(2 \times n)}^{\psi}= \left\{J\left(S_{D_{i}^{(2\times n)}}^{\psi}, S_{D_{j}^{(2\times n)}}^{\psi}\right)\mid 1 \leq i < j \leq m \right\}  $$

The adjusted *p*-values resulting from the gene set analysis of a given expression dataset are often sorted based on their adjusted *p*-values. Then gene sets with the smallest adjusted *p*-values are considered for further investigation and interpretation. Therefore, not only is the differential enrichment status of gene sets important but also the order of their significance. To assess the agreement in the order of gene sets reported as differentially enriched when analyzing replicate datasets of the same size using a method *ψ*, we use Kendall’s coefficient of concordance [24]. The Kendall’s coefficient of concordance ranges between 0 and 1, with 0 indicating no agreement and 1 indicating complete agreement, i.e. the same order of gene sets when sorted by their adjusted *p*-values. Since we aim to quantify the agreement in the order of gene sets predicted as differentially enriched across replicate datasets of the same size, we only consider gene sets that are predicted as differentially enriched for at least one replicate dataset.

Further, we compare the overlap between the results of a gene set analysis method *ψ* when applied to dataset *D*_2×*n*_ and *D*. This is done to evaluate if the dataset with the smaller sample size is enough to reproduce the results when using the whole dataset *D*. Therefore, we construct a multiset $W_{(2 \times n)}^{\psi }$ of *m* overlap scores, as follows:

3 $$  W_{(2 \times n)}^{\psi}= \left\{J\left(S_{D_{i}^{(2\times n)}}^{\psi}, S_{D}^{\psi}\right) \mid 1 \leq i \leq m \right\}  $$

High overlap scores suggest that a sample size of 2×*n* might be enough for obtaining equivalent results as those achieved using the whole dataset.

Also, to evaluate the specificity of gene set analysis methods, we conduct an additional experiment, referred to as the control-control experiment. The procedure for generating control-control replicate datasets in this experiment is similar to that of the balanced case-control replicate datasets with the sole difference being that only actual control samples are used for constructing the replicate datasets. In other words, each replicate dataset for the control-control experiment is made by random sampling (without replacement) of *n* samples from the *n*_*C*_ controls of *D* (where $n < \frac {n_{C}}{2}$) and another *n* samples from the *n*_*C*_ control samples of *D*. The former group of *n* samples are considered controls in the replicated dataset and the latter group are considered cases.

## Results

To visualize the change in reproducibility of a gene set analysis method across sample sizes, we utilize an arrangement of modified heat maps, hereafter referred to as a pine plot. Each triangular heat map in a pine plot—referred to as a layer—represents values above the diagonal in an overlap matrix (as described in “Methodology” section). Each layer visualizes the overlap scores calculated based on the results of the gene set analysis of replicate datasets of the same sample size. The colour intensity of a cell (*i*,*j*) in each layer represents $J\left (S_{D_{i}^{(2\times n)}}, S_{D_{j}^{(2\times n)}}\right)$, which is the overlap between the result of gene set analysis of two replicate datasets ($S_{D_{i}^{(2\times n)}}$ and $S_{D_{j}^{(2\times n)}}$). When *i*=*j*, a value of 1 is assigned to the cell (*i*,*i*) of the overlap matrix, which is represented with a red colour and serves as a visual reference point.

**Fig. 1:**
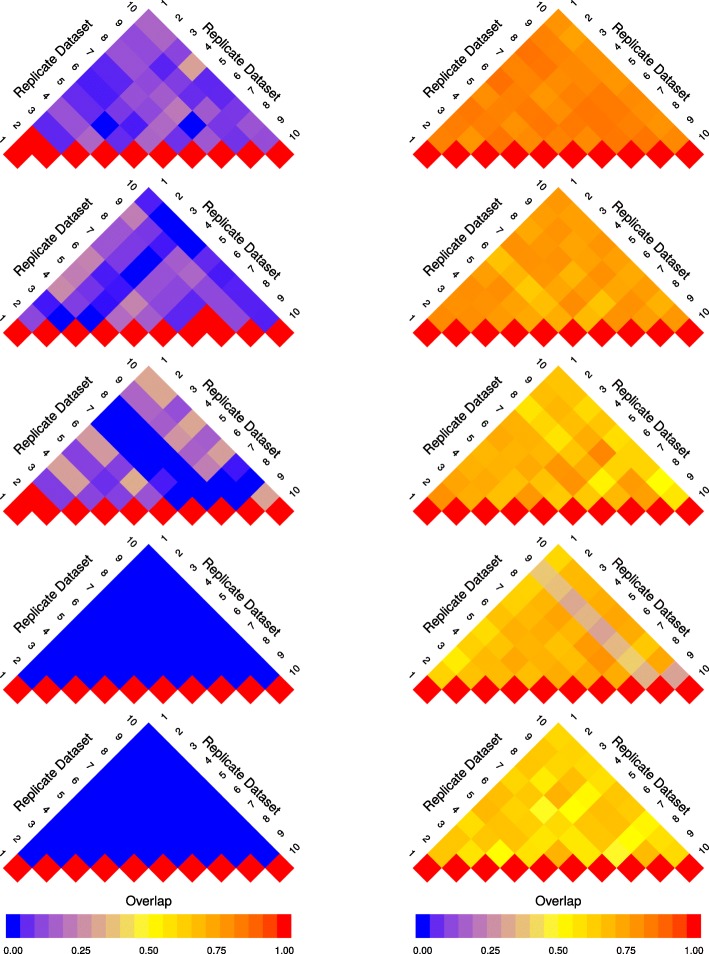
Pine plots for GSEA-G and GSEA-S (dataset GSE53757) Pine plots for dataset GSE53757 showing reproducibility of the results from GSEA-S (left) and GSEA-G (right) across sample sizes. Reproducibility is quantified by overlap score (Eq. 1). Each layer of the pine plot illustrates the overlap score of the results of a method for 10 replicate datasets with the same sample size. From top to bottom, the pine plots show replicates with sample size 2×20, 2×15, 2×10, 2×5, and 2×3. The overlap score ranges from 0 to 1 represented by a gradient from blue to red, respectively, separated by yellow in the middle (overlap of 0.5). The overlap score increases as sample size increases; however, the rate of increase for GSEA-S is lower than that of GSEA-G

**Fig. 2:**
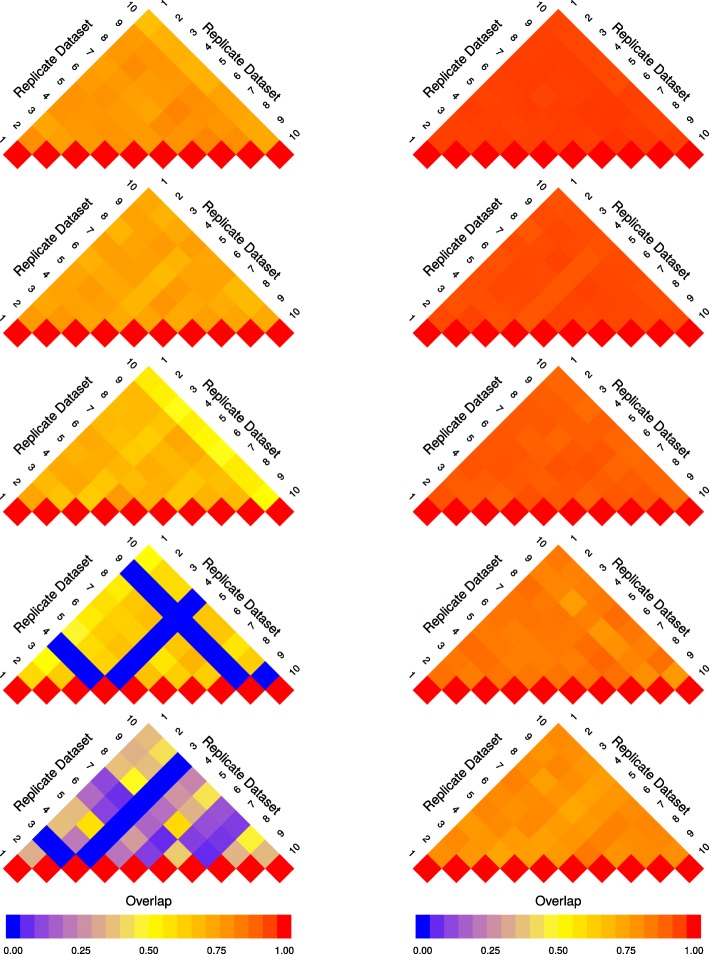
Pine plots for ORA and GAGE (dataset GSE53757) Pine plots for dataset GSE53757 showing reproducibility of the results from ORA (left) and GAGE (right). See Fig. 1 caption for more information. The pine plots suggest that the overlap between replicates is larger in comparison to that of GSEA-S and GSEA-G. GAGE has more agreement between replicates when using lower sample sizes such as 3 compared to the other methods shown (including in Fig. 1), and the overlap scores continue to improve for higher numbers of samples

The pine plots in Figs. 1 and 2 depict the change in reproducibility of results from GSEA-S, GSEA-G, ORA, and GAGE for replicate datasets of size 2×3, 2×5, 2×10, 2×15, and 2×20, where 2×*n* represents the size of a dataset with *n* controls and *n* cases. These methods were chosen as they represent the range of results shown by all 13 methods. The pine plots for the remaining methods for dataset GSE53757 are provided as Additional file 1. Visualizations and tables corresponding to datasets GSE10334 and GSE13355 are available from the authors.

The plots in Figs. 1 and 2 show that reproducibility increases as sample size increases. However, the extent of the increase in the overlap scores is not the same across all methods. GSEA-S, as depicted in Fig. 1, has lower overlap scores overall compared to GSEA-G. In Fig. 2, ORA and GAGE have higher overlap scores when using sample sizes larger than 2×10 compared to GSEA-S and GSEA-G.

The pine plots for all 13 methods illustrate the following results. PADOG, GSEA-S, and Camera show the lowest overlap scores. Pine plots for ROAST, PAGE, ORA, GSVA, and GSEA-G show a distinct transition from low overlap (for sample sizes less than or equal to 2×5) to high overlap scores (for sample sizes more than 2×10) as sample size increases. ssGSEA, GlobalTest, GAGE, and PLAGE report a large number of gene sets as differentially enriched. These methods tend to achieve high overlap scores as well.

**Fig. 3:**
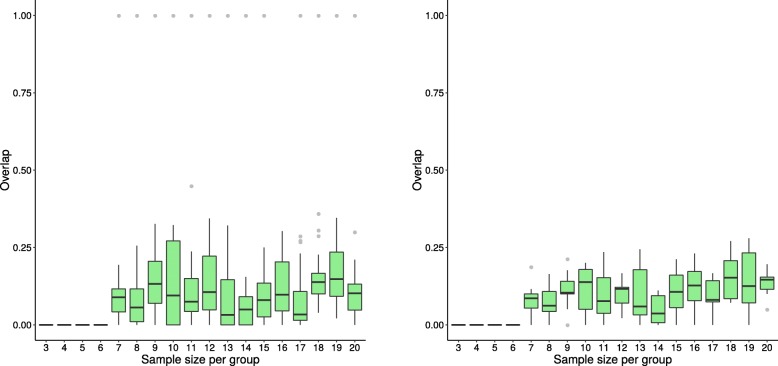
Distribution of overlap score for GSEA-S (dataset GSE53757) Box plots showing the distribution of overlap scores resulting from gene set analysis utilizing GSEA-S when using the original dataset GSE53757 for generating replicate datasets. The panel on the left shows the overlap scores from replicate datasets, while that on the right depicts the overlap scores of each replicate dataset and the whole dataset. Dataset sample sizes are 2×*n* (3≤*n*≤20), where *n* is the sample size per group. The x-axis shows *n*, the sample size per group, and the y-axis shows the overlap scores

**Fig. 4:**
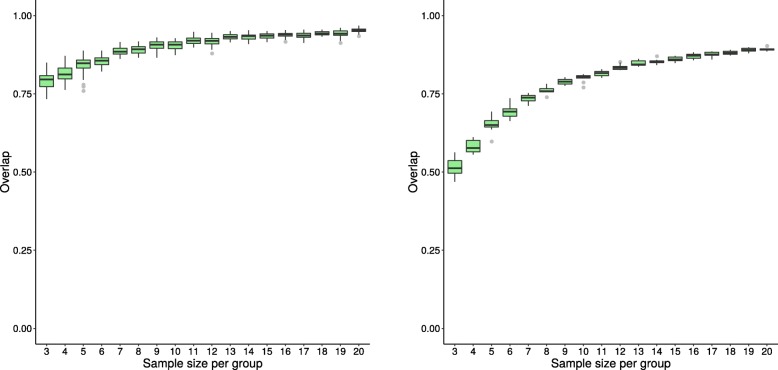
Distribution of overlap score for GAGE (dataset GSE53757) Box plots showing the distribution of overlap scores resulting from gene set analysis utilizing GAGE when using the original dataset GSE53757 for generating replicate datasets. The panel on the left shows the overlap scores from replicate datasets, while that on the right depicts the overlap scores of each replicate dataset and the whole dataset. See Fig. 3 caption for more information

**Fig. 5:**
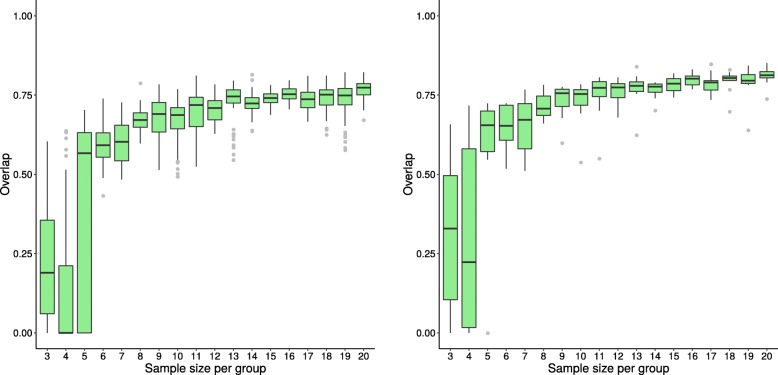
Distribution of overlap score for ORA (dataset GSE53757) Box plots showing the distribution of overlap scores resulting from gene set analysis utilizing ORA when using the original dataset GSE53757 for generating replicate datasets. The panel on the left shows the overlap scores from replicate datasets, while that on the right depicts the overlap scores of each replicate dataset and the whole dataset. See Fig. 3 caption for more information

The box plots in Figs. 3, 4, and 5 illustrate the distribution of the overlap scores across sample sizes when the results of the replicate datasets are compared to each other (calculated using Eq. 1), as well as when the results of replicate datasets are compared to the results using the entire dataset, i.e. original dataset (calculated using Eq. 3). Figures 3, 4, and 5 show representative results from three methods; plots for the remaining methods are in Additional file 1. For all methods except GSEA-S and PADOG, the agreement between overlap scores increases as sample size increases.

To statistically assess if there is a significant difference between the overlap scores across sample sizes, i.e. $P_{(2 \times 3)}^{\psi }, \dots, P_{(2 \times 20)}^{\psi }$, for each method, a Kruskal-Wallis test was used. The *p*-values resulting from these tests, shown in Additional file 1: Table S1, suggest that the overlap scores significantly vary across sample sizes irrespective of the original dataset being used.

**Fig. 6:**
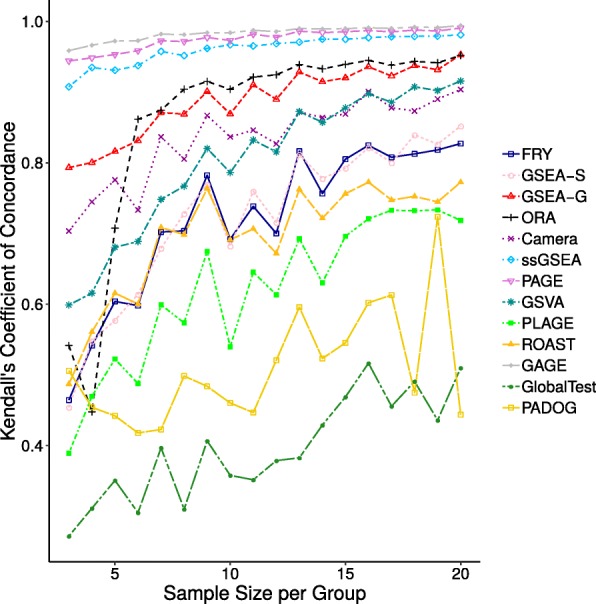
Kendall’s coefficient of concordance results for dataset GSE53757 Kendall’s coefficient of concordance for each method under study when using the original dataset GSE53757 for generating replicate datasets. The x-axis shows the sample size. The y-axis shows concordance coefficients of the results of gene set analysis of 10 replicate datasets of the same size

Figure 6 depicts Kendall’s concordance coefficients for replicate datasets across sample sizes for all methods under study. The figure illustrates that concordance coefficient increases as the sample size increases.

**Fig. 7:**
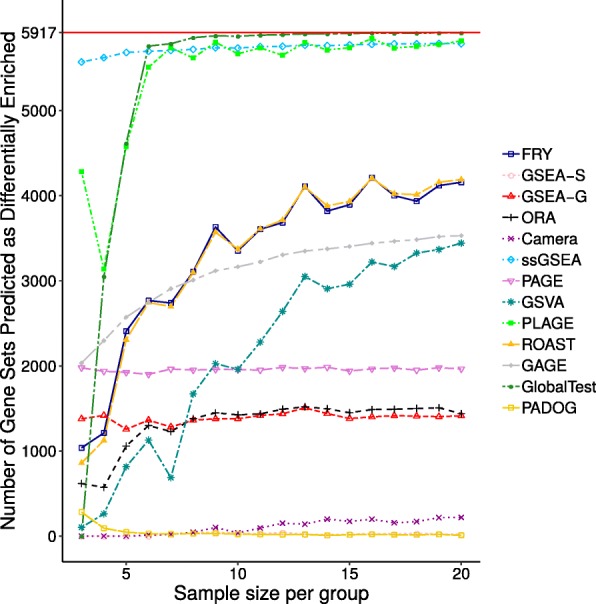
Number of gene sets reported as differentially enriched for dataset GSE53757 The number of gene sets predicted as differentially enriched for each method under study when using the original dataset GSE53757 for generating replicate datasets. The x-axis shows the sample size per group. The y-axis shows the average number of gene sets predicted as differentially enriched across 10 replicate datasets of the same size. The red line parallel to the x-axis shows the size of the gene set database being used, i.e. the maximum possible number of gene sets that could be predicted as being differentially enriched

Figure 7 illustrates the average number of gene sets reported as differentially enriched across sample sizes. PADOG, GSEA-S, and Camera tend to report a small number of differentially enriched gene sets compared to the other methods. The number of gene sets reported as differentially enriched by GAGE, GSVA, ROAST, and FRY substantially increases as sample size increases, while the rest of the methods reach an almost constant number of gene sets predicted as being differentially enriched. Visualizations analogous to Figs. 6 and 7 for datasets GSE10334 and GSE13355 are provided in Additional file 1. These Figures, as well as the pine plots and box plots for these datasets, showed patterns consistent with those for dataset GSE53757.

The control-control experiment was conducted using dataset GSE53757. Additional file 1: Table S2 shows the average number of differentially enriched gene sets across sample sizes in the control-control experiment for all of the methods. Since there is no true differential enrichment expected in the control-control experiment, the average values in this table represent the average number of false positives. ssGSEA, GAGE, PAGE, and PADOG are methods with non-zero false positive counts across sample sizes. ssGSEA results in the largest number of false positives followed by GAGE, and then PAGE. Also, increasing sample size for these methods does not reduce the frequency of false positives. Surprisingly, an increase in sample size leads to an increase in false positives when using GAGE. The number of false positives reported by PADOG decreases rapidly as sample size increases, and for sample sizes larger than 2×5, it only reports a small number of false positives. Camera reports almost no false positives for sample sizes less than 2×9, but it reports a small number of false positives as sample size increases. ORA, GSVA, GlobalTest, PLAGE, ROAST, and FRY rarely, if ever, report any false positives.

## Discussion

Reproducibility of the results of gene set analysis methods, as in any other scientific context, is an essential condition for having confidence in the methods. In this research, we applied a systematic approach for quantitatively assessing the reproducibility of gene set analysis methods. By using real expression datasets, the proposed approach strives for a realistic assessment of the reproducibility of gene set analysis methods across sample sizes. We also measure the specificity of gene set analysis methods as a complement to the reproducibility assessment.

To visualize overlap between the results of a gene set analysis method across sample sizes, we introduced and used pine plots. The utility of pine plots, however, is not limited to this application. In general, pine plots can be used to visualize the interaction between several variables defined using a symmetric function while controlling for potentially confounding factors. In practice, most functions for measuring the interaction between variables are symmetric functions—for example, Pearson correlation and Spearman’s rank correlation coefficients. Also, symmetry is a necessary condition for any well-defined metric or distance function [25]. Therefore, the symmetric condition does not limit the usability of pine plots. The pine plots in this paper illustrated a general increase in reproducibility as sample size increases. While boxplots can only show the distribution of overlap scores, pine plots are capable of showing the extent of the overlap between each pair of replicate datasets, further highlighting the reproducibility of replicate datasets of the same size.

The reproducibility of gene set analysis across replicate datasets is a necessary condition for obtaining biologically meaningful results, but not a sufficient one. To illustrate, consider a hypothetical method that always predicts all of the gene sets in a gene set database as differentially enriched regardless of the phenotype being examined. Obviously, the results of this method are perfectly reproducible (always an overlap score of 1). However, such a method produces a large number of false positives—i.e., suffers from a lack of specificity—and, as a consequence, does not provide any biological insight. Therefore, we also investigated the specificity of gene set analysis methods. Gene set analysis methods that tend to predict very few gene sets as differentially enriched achieve high specificity. However, these methods often suffer from a lack of sensitivity. In this research, we used the number of differentially enriched gene sets predicted by each method to reveal such scenarios.

The pine plots and box plots showed an increase in reproducibility as sample size increases. However, the extent of reproducibility and the rate at which it grows by sample size was different across methods.

We evaluated reproducibility not only based on the differential enrichment of gene sets but also on the order in which they are predicted, where the order is defined using significance values of gene sets. Kendall concordance coefficients were used to determine if the gene sets predicted as significantly differentially enriched by each method are consistently reported in the same order across replicated datasets of the same sample size.

This research provides insights into the behaviour of specific gene set analysis methods. For instance, GSEA-S achieves low overlap scores across replicate datasets, even for larger sample sizes such as 2×20. Meanwhile no differentially enriched gene sets are predicted by this method using small sample sizes (see Figs. 1, 3, and 7). GSEA-S was expected to predict few differentially enriched gene sets using small sample sizes since large sample sizes are required for this method to assess significance based on sample permutation. For example, for an experiment with 3 control and 3 case samples, 20 distinct sample permutations exist—the combination of 3 out of 6. Therefore, the smallest non-zero *p*-value is 0.05, which we did not consider significant. For a sample size of 2×10 or higher, GSEA-S predicts an average of 15 gene sets as differentially enriched (Fig. 7). This number remains steady while Kendall’s concordance increases (Fig. 6), which suggests that 2×10 samples might be a reasonable lower bound for using GSEA-S.

PADOG, which has been designed to take gene set overlap into account and increase specificity, has low overlap scores between replicate datasets even for the largest sample sizes considered. Like GSEA-S and Camera, PADOG also predicts few gene sets as differentially enriched. Lack of reproducibility across large sample sizes for both methods may suggest that the gene sets predicted as differentially enriched are false positives, i.e. gene sets incorrectly predicted as being differentially enriched.

PLAGE, ssGSEA, and GlobalTest tend to report nearly all gene sets as differentially enriched. This is always the case for ssGSEA regardless of the sample size of the replicate datasets used. For small sample sizes, PLAGE and GlobalTest report fewer gene sets as differentially enriched, but this number rapidly increases for larger sample sizes. Furthermore, PLAGE appears to be more sensitive to the dataset used since it predicts far fewer gene sets as differentially enriched for the dataset with higher variability across case and control samples, as with GSE10334 (see Additional file 1: Figures S2 and S21). The number of gene sets predicted explains why these methods achieve high overlap scores. Since it is unlikely that such a large number of gene sets are differentially enriched in a living organism, we assume that these methods also predict a large number of false positives.

Given the above, relying on gene sets predicted as being differentially enriched by GlobalTest, PLAGE or ssGSEA may lead to interpretations that are incorrect or biased towards a hypothesis of interest. However, the most statistically significant gene sets, i.e. gene sets with the lowest adjusted *p*-value, suggested by these methods may still be biologically relevant. Therefore, we suggest further research be conducted to evaluate how best to use these methods and interpret their results.

GlobalTest’s and PLAGE’s relatively low Kendall concordance coefficients depicted in Fig. 6 show that the order in which they predict the differentially enriched gene sets is not conserved across replicate datasets. For PLAGE this may be explained by considering that for each gene set, the method defines “the activity level in terms of the first eigenvector, ‘metagene’, in the singular value decomposition" [13]. By ignoring other eigenvectors of an expression profile for a gene set, it cannot completely capture the variability of expression of genes within a gene set. This means that the gene sets predicted as being differentially enriched by PLAGE could show variation in statistical significance across replicate datasets, and therefore, be ranked differently for each replicate dataset.

For sample sizes larger than 2×5, ORA remains consistent in the number of gene sets reported as differentially enriched suggesting that 2×5 is a reasonable lower bound for sample sizes when using ORA. PAGE also shows a behaviour similar to that of ORA. Since PAGE and ORA are parametric methods and their calculated gene set scores are a function of the number of differentially expressed genes, this behaviour was expected.

When replicate datasets of various sizes are generated from one original dataset, it is expected that a gene set analysis method analyzing these replicate datasets will report approximately the same number of differentially enriched gene sets. However, this is not the case with GAGE, GSVA, FRY, and ROAST. A dramatic increase in the number of gene sets predicted as being differentially enriched was observed for these methods as sample size increases. This increase may also be partially responsible for the observed increase in the overlap scores of these methods as sample size increases.

FRY and ROAST closely mirror each other in the number of gene sets predicted as being differentially enriched as well as their Kendall concordance coefficients across sample sizes. As FRY was designed to be a fast approximation of ROAST, this behaviour is understandable. However, since these methods, as well as GAGE and GSVA, report large numbers of gene sets as being differentially enriched, we assume that these methods may lead to more false positives as sample size increases.

Measuring sensitivity using real datasets is challenging, if not impossible, as the differential enrichment status of gene sets for real datasets are not known. Simulated data, on the other hand, often suffer from oversimplified assumptions such as constant or zero gene-gene correlation or normally distributed expression values [26–28] leading to biased evaluations of gene set analysis methods [29]. We suggest development of standard synthesized datasets without relying on such assumptions as future research in the community. This would alleviate the challenges caused by the lack of gold standard datasets for the evaluation of gene set analysis methods. A methodology such as the one utilized in this paper could be used for such standard datasets.

The results of the control-control experiment indicate that some methods suffer from a lack of specificity, even in the absence of differential expression. It would be expected that as the number of samples increases, the number of false positives reported would decrease, i.e. specificity increases; however, it is not the case for some methods. As depicted in Additional file 1: Table S2, GAGE and ssGSEA report the highest number of false positives. Almost all gene sets are predicted by ssGSEA as differentially enriched in both the control-control experiment and case-control experiments (see Fig. 7 and also in Additional file 1: Figures S21 and S22), regardless of the sample size. GAGE reports a large number of false positives as sample size increases. PAGE and GSEA-G also report a large number of false positives, but this number remains relatively consistent and is not affected by the sample size used. Therefore, increasing the sample size is not a viable solution for improving the results of these methods. PADOG reports fewer false positives as sample size increases; however, PADOG also reports a small number of differentially enriched gene sets in the case-control experiments as well. This shows that although PADOG achieves a high specificity, it may suffer from a lack of sensitivity, as the reported false positives might overwhelm the results. GSEA-S and Camera have a similar issue with being overwhelmed with false positives since they also report a small number of gene sets as differentially enriched. GSVA, Globaltest, FRY, and ROAST do not appear to report false positives in the control-control experiment regardless of sample size being used. However, this control-control experiment measures the number of false positive in the absence of differential expression and does not say anything about the specificity of these methods in the presence of differential expression. The large number of gene sets predicted as differentially enriched in the case-control experiments, as depicted in Fig. 7 and also in Additional file 1: Figures S21 and S22, suggests that these methods suffer from a lack of specificity in the presence of differential expression, as such a drastic change in gene expression is unlikely for a living organism. ORA, as expected, did not report any false positive in the absence of differentially expressed genes. It also reported a smaller number of differentially enriched gene sets in the case-control experiments—in comparison to GSVA, Globaltest, FRY, and ROAST. However, it still reported a substantially large number of differentially enriched gene sets for each case-control experiment (almost 20% of gene sets in $\mathbb {G}$). This suggests that ORA could still suffer from lack of specificity in the presence of differential expression though not to the degree of the other methods such as GSVA, Globaltest, FRY, and ROAST. This can be attributed to the presence of gene set overlap [30].

In this paper, we evaluated the results of gene set analysis methods using balanced datasets, i.e. datasets with the same number of cases and controls. While we expect consistent results for datasets that are not drastically imbalanced, we suggest investigating the effect of dataset imbalance on the results of gene set analysis methods.

## Conclusion

The systematic methodology described in this paper can be successfully used to evaluate the reproducibility of results from gene set analysis methods, allowing comparison across methods and sample sizes. The methodology was employed to evaluate the reproducibility of 13 widely used gene set analysis methods. The proposed methodology also made it possible to measure the specificity of these methods using real datasets. From the results we conclude that, as a general pattern, reproducibility, as measured by an overlap score, increases with sample size. However, the rate of increase is method-dependent. Our findings suggest that for all methods in the study, achieving reproducible results using small sample sizes—such as 3, 4, or 5 samples per group—is unlikely. However, we observed that increasing sample size is not a panacea for achieving biologically reliable results, as for some methods it decreased the specificity, i.e. introduced more false positives.

Results from this paper can aid researchers in making a choice among common gene set analysis methods for their work.

## Additional file

Additional file 1 This file includes the results of the analysis for the datasets and methods not presented in the main body of the paper. (PDF 435 kb)

## Abbreviations

GEO: Gene Expression Omnibus
GSEA-G: GSEA gene permutation
GSEA-S: GSEA sample permutation
ORA: Over-representation analysis
