# Supplementary material for: Size matters: how sample size affects the reproducibility and specificity of gene set analysis
Source: Hum Genomics. 2019 Oct 22;13(Suppl 1):42. doi: 10.1186/s40246-019-0226-2 (PMC6805317; doi:10.1186/s40246-019-0226-2)
Supplement: Supplementary file 1 — This file includes the results of the analysis for the datasets and methods not presented in the main body of the paper. (PDF 435 kb) [file 40246_2019_226_MOESM1_ESM.pdf]

**Table S1** Kruskal-Wallis test results show that there is a statistically significant difference between the reproducibility of gene set analysis methods across sample sizes for all three original datasets.

| Method | GSE53757 | GSE13355 | GSE10334 |
|---|---|---|---|
| FRY | 6.28e-13 | 2.99e-26 | 2.60e-13 |
| GSEA-S | 1.07e-11 | 1.45e-15 | 4.72e-05 |
| GSEA-G | 5.99e-12 | 6.72e-19 | 3.58e-13 |
| ORA | 5.03e-18 | 2.39e-19 | 1.67e-14 |
| Camera | 1.81e-19 | 2.11e-20 | 9.74e-05 |
| ssGSEA | 4.71e-25 | 8.70e-26 | 1.87e-26 |
| PAGE | 1.81e-16 | 5.26e-20 | 1.50e-10 |
| GSVA | 5.30e-20 | 7.17e-27 | 4.21e-07 |
| PLAGE | 2.10e-05 | 4.88e-06 | 4.89e-03 |
| ROAST | 1.37e-14 | 1.80e-26 | 1.10e-13 |
| GAGE | 2.34e-28 | 3.37e-28 | 4.51e-27 |
| GlobalTest | 6.73e-21 | 7.29e-25 | 2.58e-16 |
| PADOG | 7.10e-06 | 1.79e-17 | 7.18e-01 |

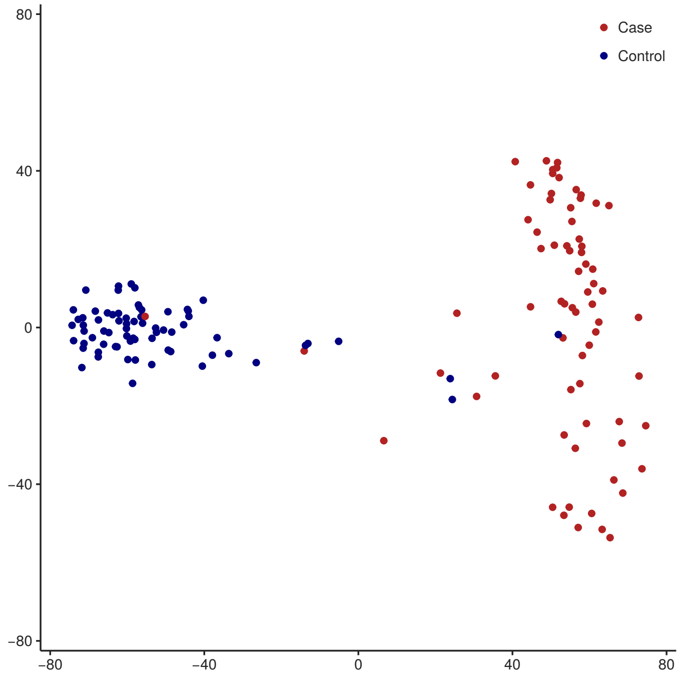


**Figure S1** MDS plot showing the relation between samples in dataset GSE53757. Each sample is represented as a point on the plot. The control samples are coloured in dark red and the case samples are coloured in blue.

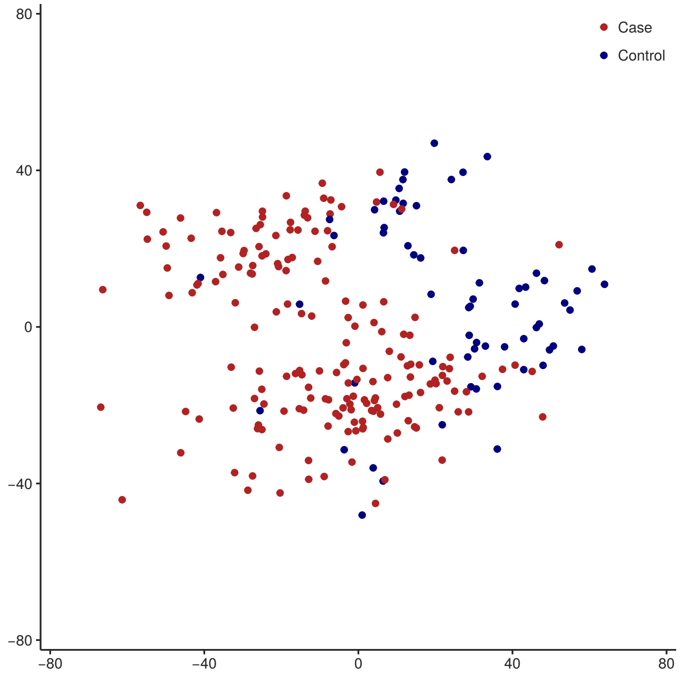


**Figure S2** MDS plot showing the relation between samples in dataset GSE10334. Each sample is represented as a point on the plot. The control samples are coloured in dark red and the case samples are coloured in blue.

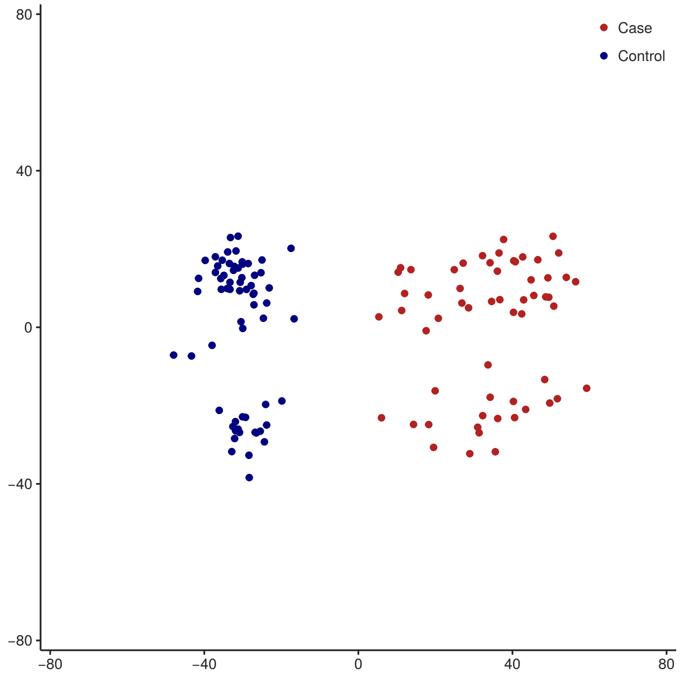


**Figure S3** MDS plot showing the relation between samples in dataset GSE13355. Each sample is represented as a point on the plot. The control samples are coloured in dark red and the case samples are coloured in blue.

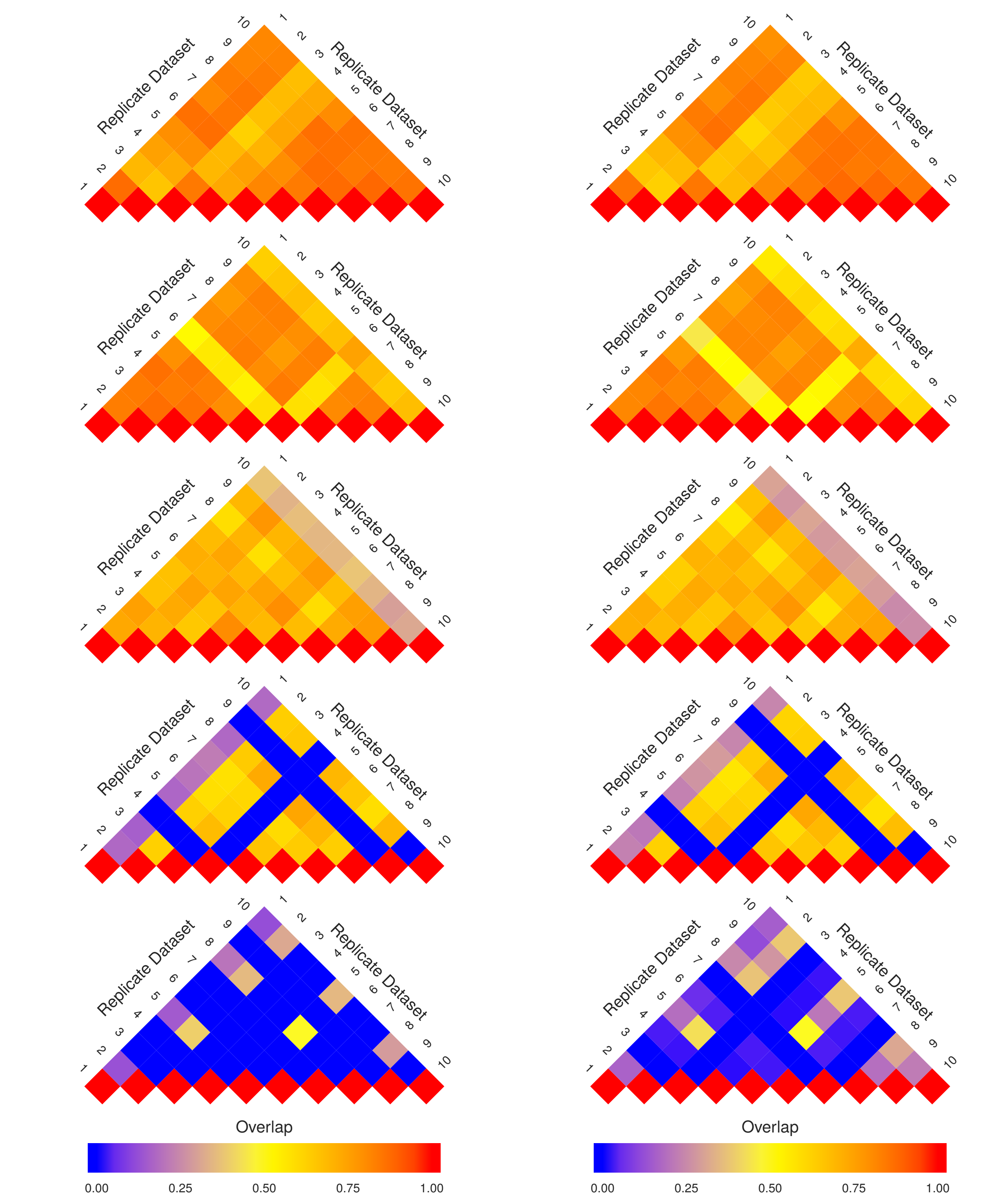


**Figure S4** Pine plots for dataset GSE53757 showing reproducibility of the results from ROAST (left) and FRY (right) across sample sizes. Reproducibility is quantified by overlap score (Equation 1). Each layer of the pine plot illustrates the overlap score of the results of a method for 10 replicate datasets with the same sample size. From top to bottom, the pine plot shows replicates with sample size $2 \times 20$, $2 \times 15$, $2 \times 10$, $2 \times 5$, and $2 \times 3$. The overlap score ranges from 0 to 1 represented by a gradient from blue to red, respectively, separated by yellow in the middle (overlap of 0.5).

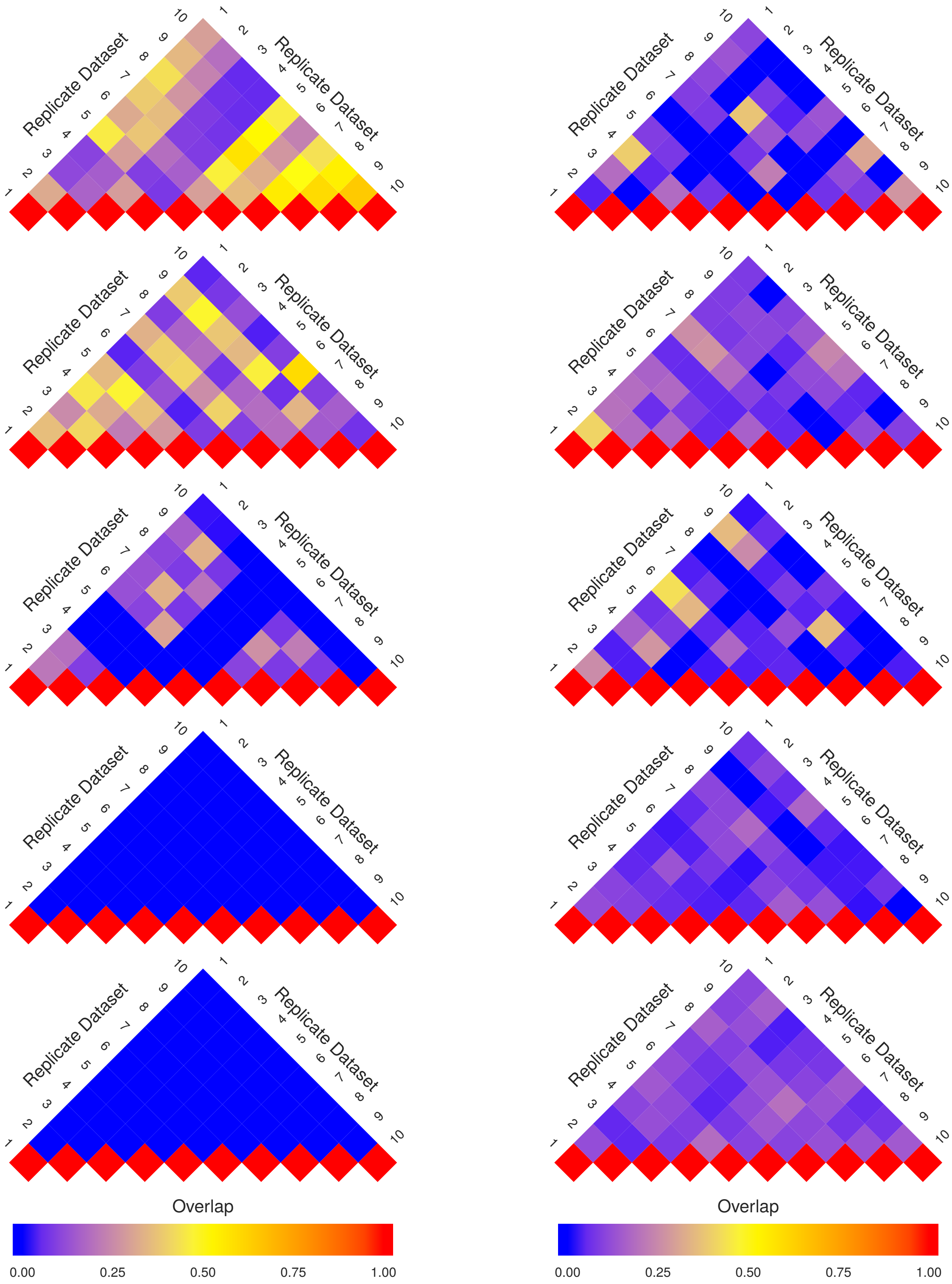


**Figure S5** Pine plots for dataset GSE53757 showing reproducibility of the results from Camera (left) and PADOG (right) across sample sizes. Reproducibility is quantified by overlap score (Equation 1). Each layer of the pine plot illustrates the overlap score of the results of a method for 10 replicate datasets with the same sample size. From top to bottom, the pine plot shows replicates with sample size $2 \times 20$, $2 \times 15$, $2 \times 10$, $2 \times 5$, and $2 \times 3$. The overlap score ranges from 0 to 1 represented by a gradient from blue to red, respectively, separated by yellow in the middle (overlap of 0.5).

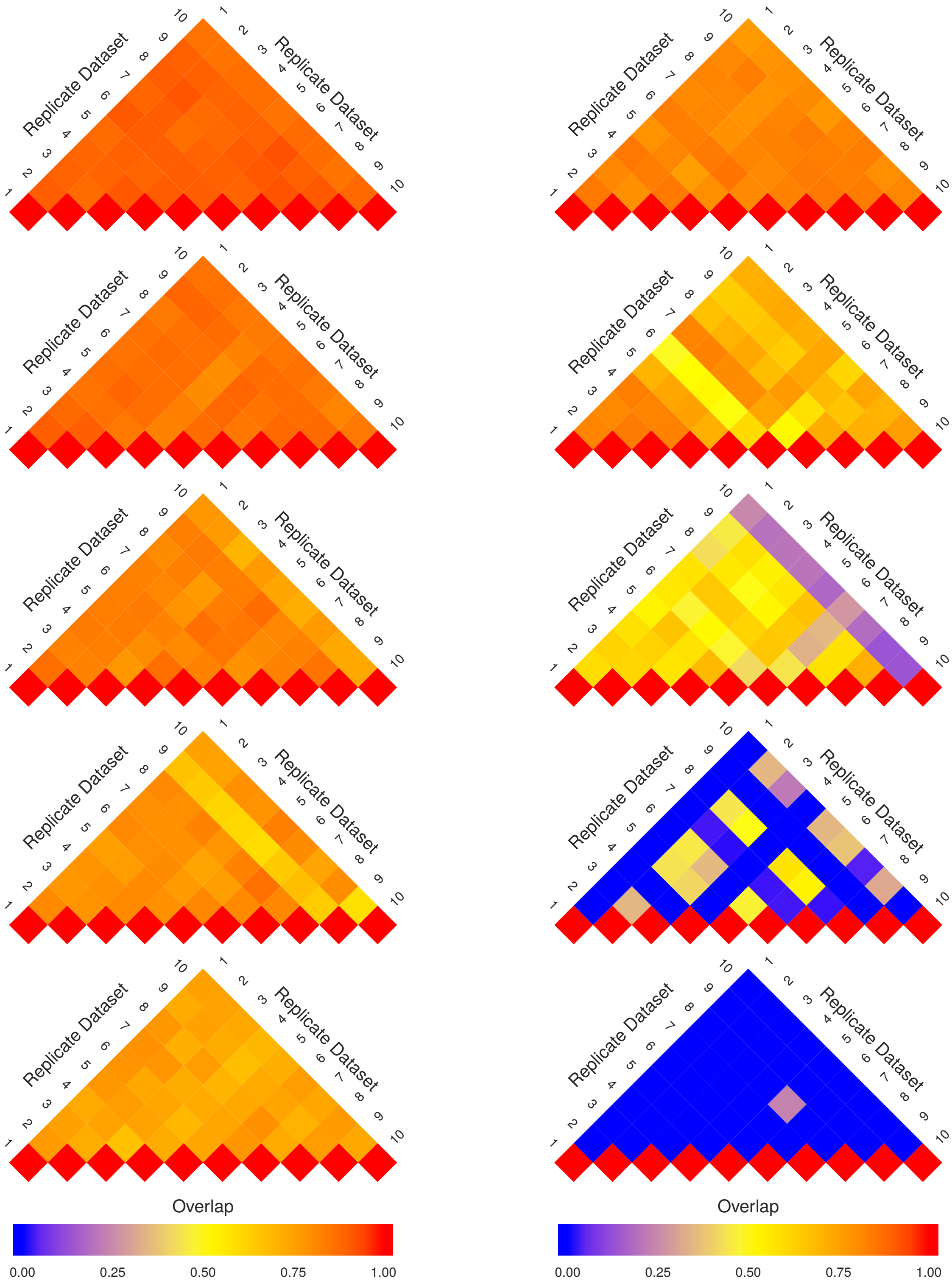


**Figure S6** Pine plots for dataset GSE53757 showing reproducibility of the results from PAGE (left) and GSVA (right) across sample sizes. Reproducibility is quantified by overlap score (Equation 1). Each layer of the pine plot illustrates the overlap score of the results of a method for 10 replicate datasets with the same sample size. From top to bottom, the pine plot shows replicates with sample size $2 \times 20$, $2 \times 15$, $2 \times 10$, $2 \times 5$, and $2 \times 3$. The overlap score ranges from 0 to 1 represented by a gradient from blue to red, respectively, separated by yellow in the middle (overlap of 0.5).

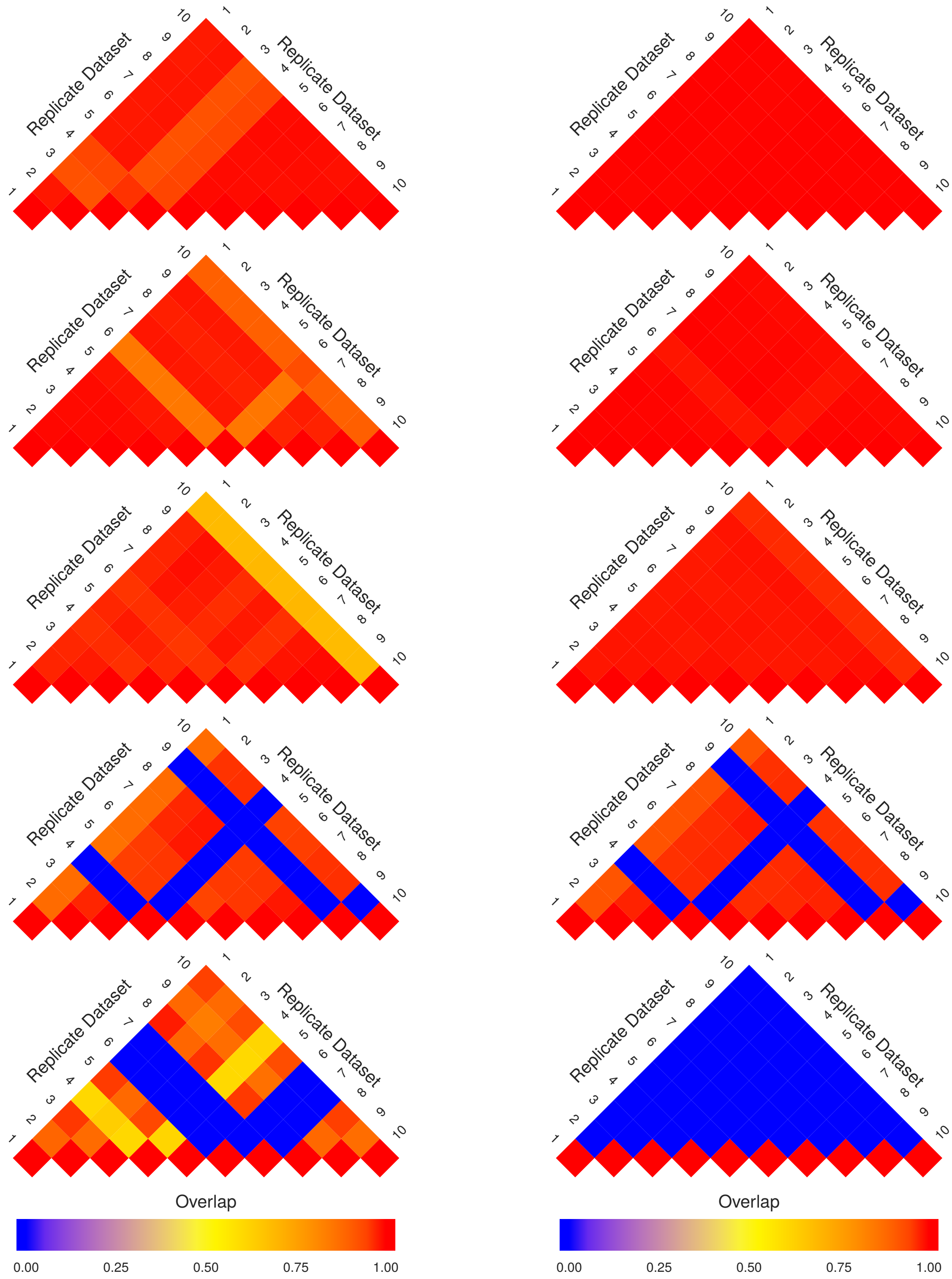


**Figure S7** Pine plots for dataset GSE53757 showing reproducibility of the results from PLAGE (left) and GlobalTest (right) across sample sizes. Reproducibility is quantified by overlap score (Equation 1). Each layer of the pine plot illustrates the overlap score of the results of a method for 10 replicate datasets with the same sample size. From top to bottom, the pine plot shows replicates with sample size $2 \times 20$, $2 \times 15$, $2 \times 10$, $2 \times 5$, and $2 \times 3$. The overlap score ranges from 0 to 1 represented by a gradient from blue to red, respectively, separated by yellow in the middle (overlap of 0.5).

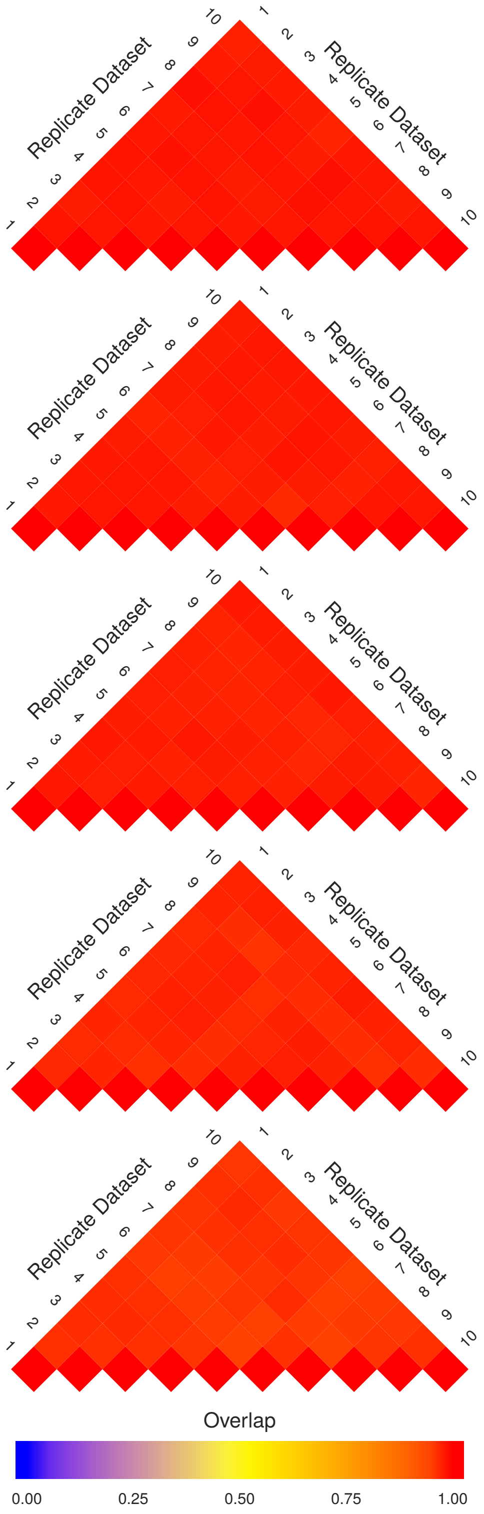


**Figure S8** Pine plots for dataset GSE53757 showing reproducibility of the results from ssGSEA across sample sizes. Reproducibility is quantified by overlap score (Equation 1). Each layer of the pine plot illustrates the overlap score of the results of a method for 10 replicate datasets with the same sample size. From top to bottom, the pine plot shows replicates with sample size $2 \times 20$, $2 \times 15$, $2 \times 10$, $2 \times 5$, and $2 \times 3$. The overlap score ranges from 0 to 1 represented by a gradient from blue to red, respectively, separated by yellow in the middle (overlap of 0.5).

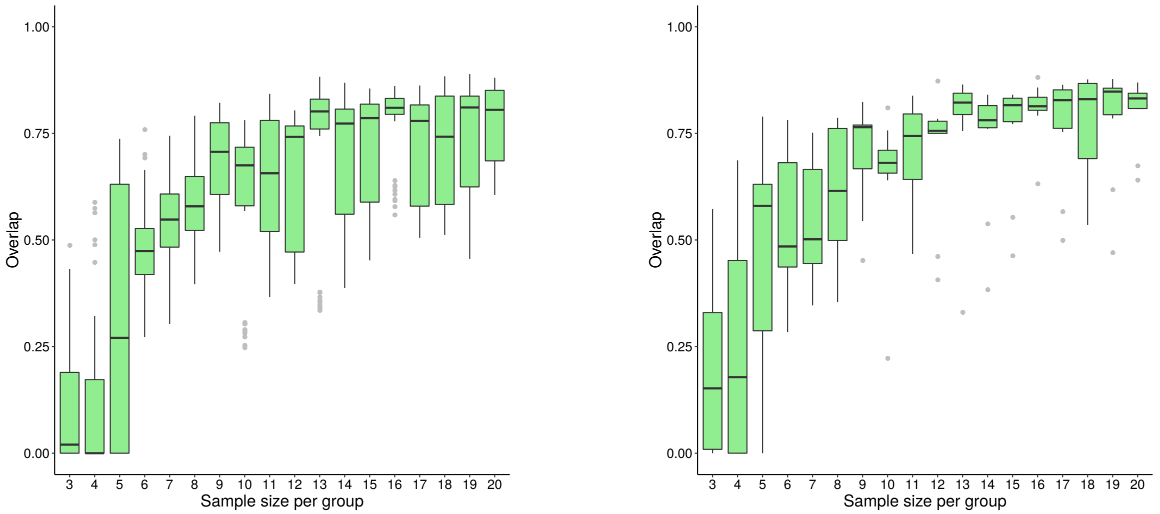


**Figure S9** Box plots showing the distribution of overlap scores resulting from gene set analysis using FRY when using the original dataset GSE53757 for generating replicate datasets. The panel on the left shows the overlap scores from replicate datasets, while that on the right depicts the overlap scores of each replicate dataset and the whole dataset. See Figure 3 caption for more information.

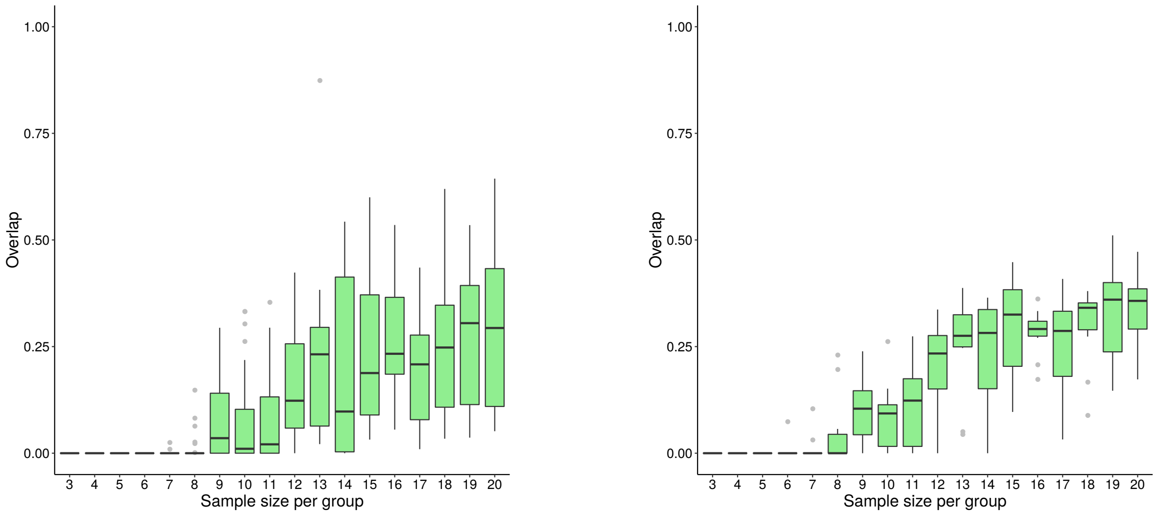


**Figure S10** Box plots showing the distribution of overlap scores resulting from gene set analysis using Camera when using the original dataset GSE53757 for generating replicate datasets. The panel on the left shows the overlap scores from replicate datasets, while that on the right depicts the overlap scores of each replicate dataset and the whole dataset. See Figure 3 caption for more information.

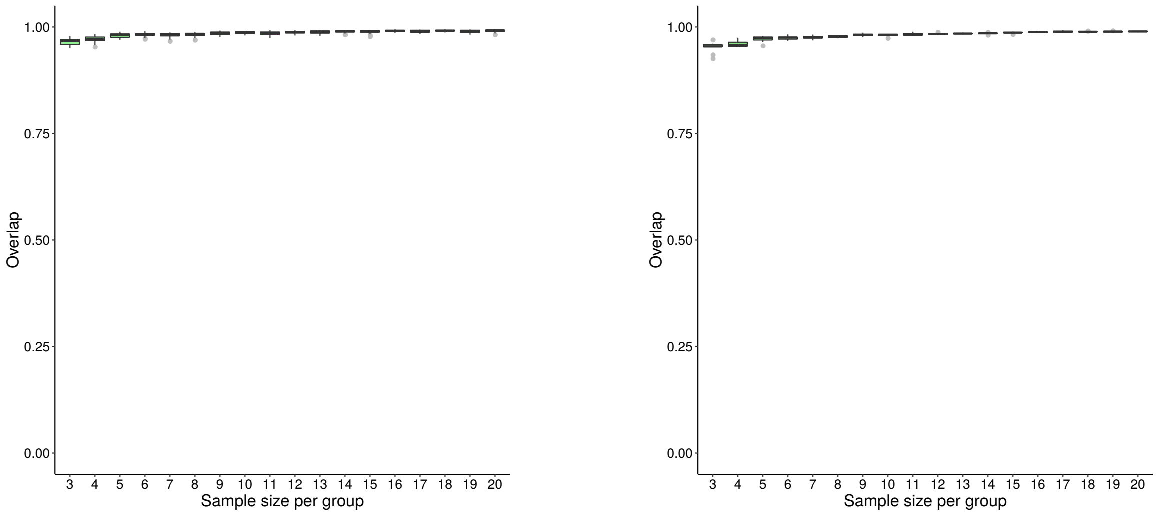


**Figure S11** Box plots showing the distribution of overlap scores resulting from gene set analysis using ssGSEA when using the original dataset GSE53757 for generating replicate datasets. The panel on the left shows the overlap scores from replicate datasets, while that on the right depicts the overlap scores of each replicate dataset and the whole dataset. See Figure 3 caption for more information.

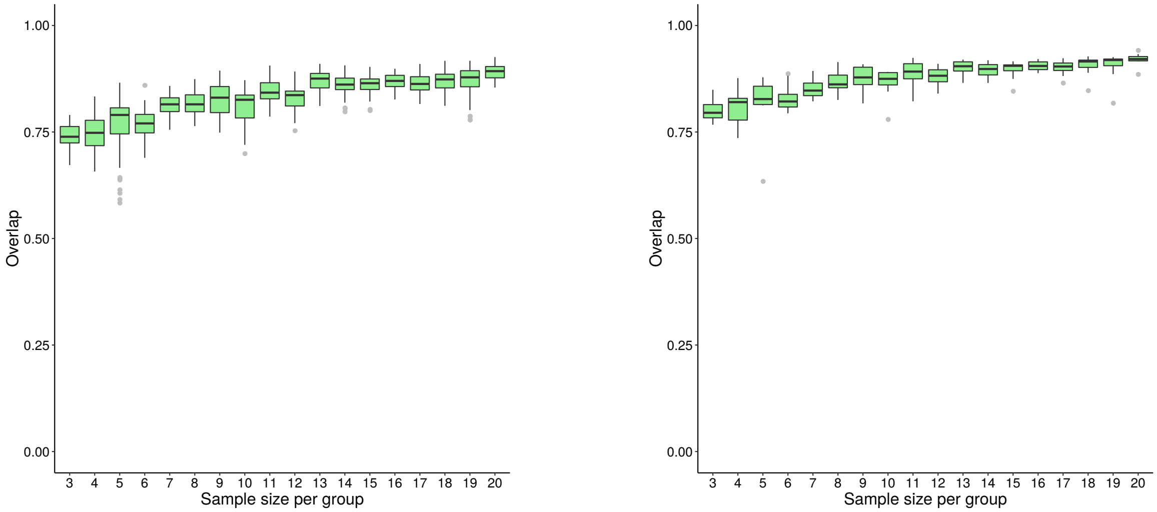


**Figure S12** Box plots showing the distribution of overlap scores resulting from gene set analysis using PAGE when using the original dataset GSE53757 for generating replicate datasets. The panel on the left shows the overlap scores from replicate datasets, while that on the right depicts the overlap scores of each replicate dataset and the whole dataset. See Figure 3 caption for more information.

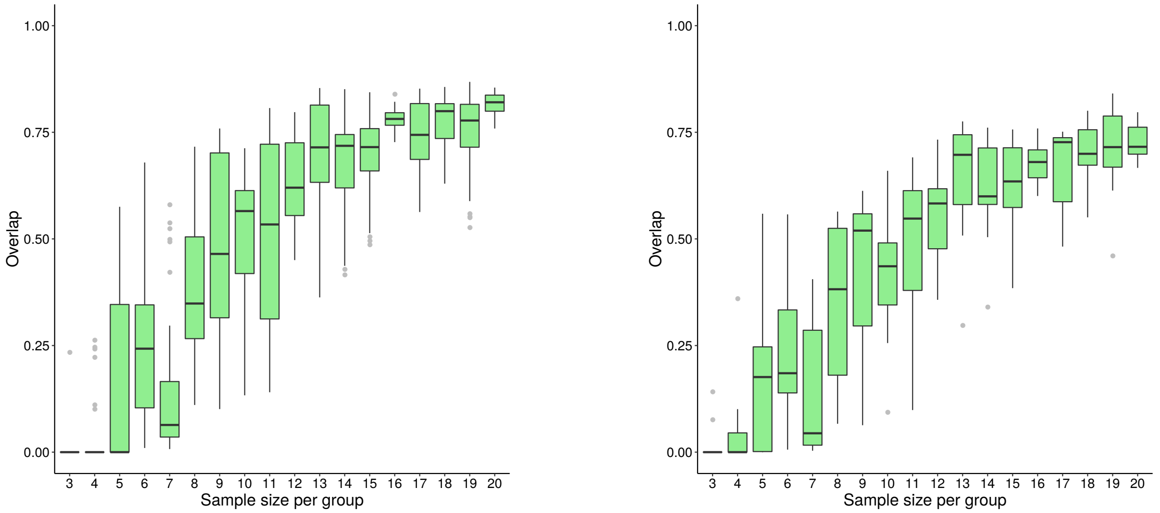


**Figure S13** Box plots showing the distribution of overlap scores resulting from gene set analysis using GSVA when using the original dataset GSE53757 for generating replicate datasets. The panel on the left shows the overlap scores from replicate datasets, while that on the right depicts the overlap scores of each replicate dataset and the whole dataset. See Figure 3 caption for more information.

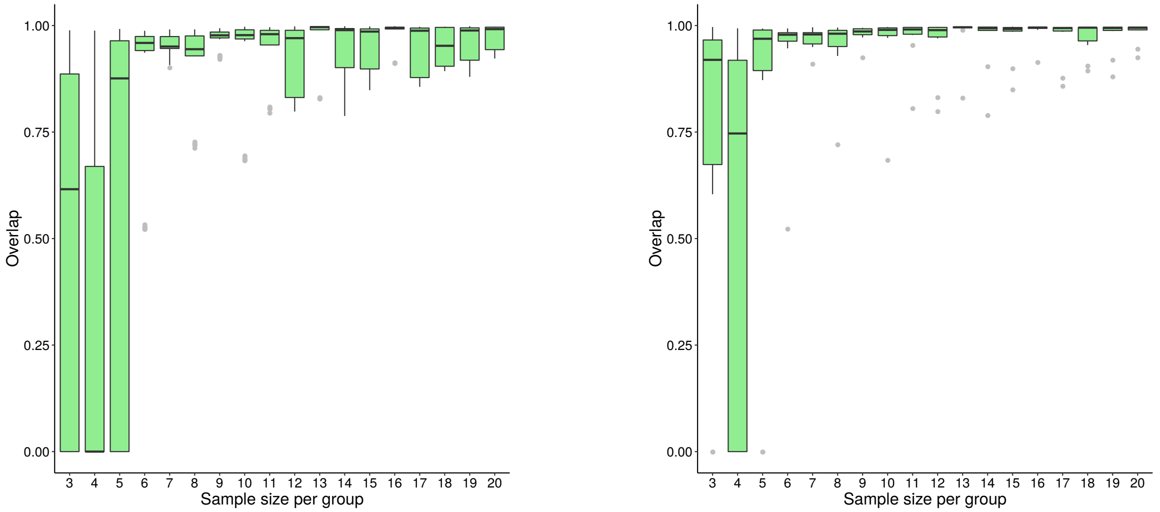


**Figure S14** Box plots showing the distribution of overlap scores resulting from gene set analysis using PLAGE when using the original dataset GSE53757 for generating replicate datasets. The panel on the left shows the overlap scores from replicate datasets, while that on the right depicts the overlap scores of each replicate dataset and the whole dataset. See Figure 3 caption for more information.

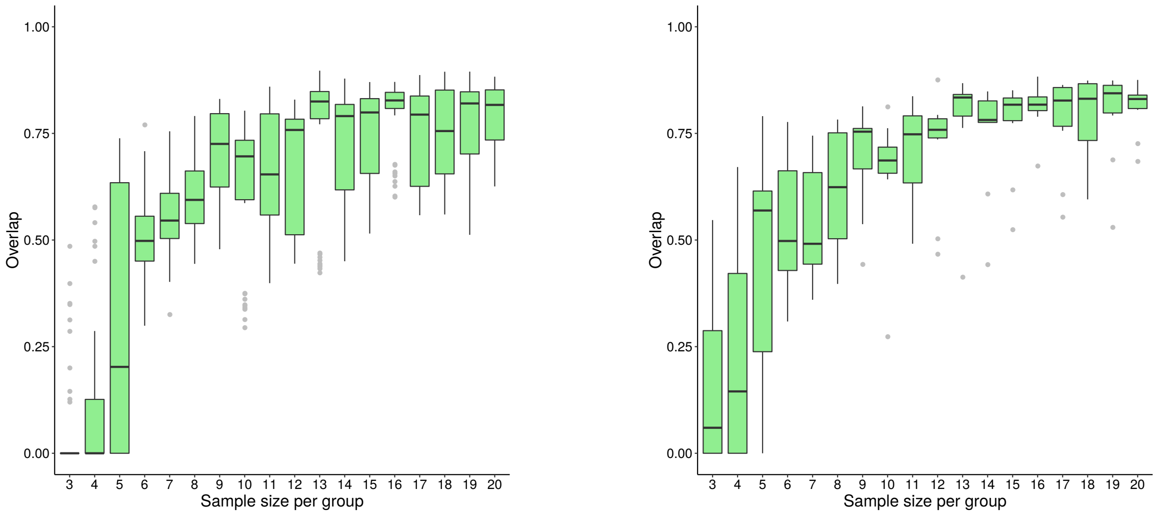


**Figure S15** Box plots showing the distribution of overlap scores resulting from gene set analysis using ROAST when using the original dataset GSE53757 for generating replicate datasets. The panel on the left shows the overlap scores from replicate datasets, while that on the right depicts the overlap scores of each replicate dataset and the whole dataset. See Figure 3 caption for more information.

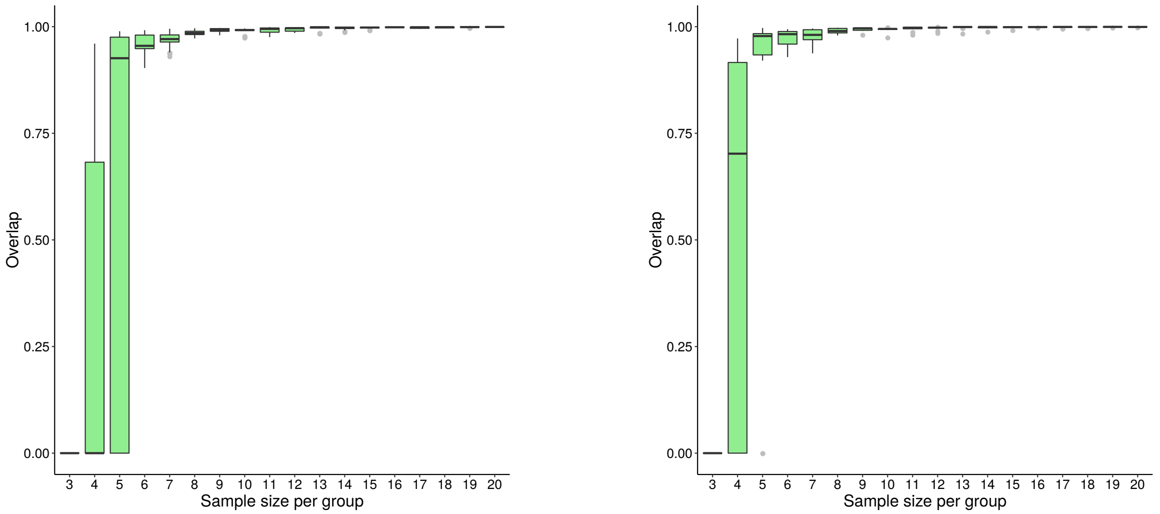


**Figure S16** Box plots showing the distribution of overlap scores resulting from gene set analysis using GlobalTest when using the original dataset GSE53757 for generating replicate datasets. The panel on the left shows the overlap scores from replicate datasets, while that on the right depicts the overlap scores of each replicate dataset and the whole dataset. See Figure 3 caption for more information.

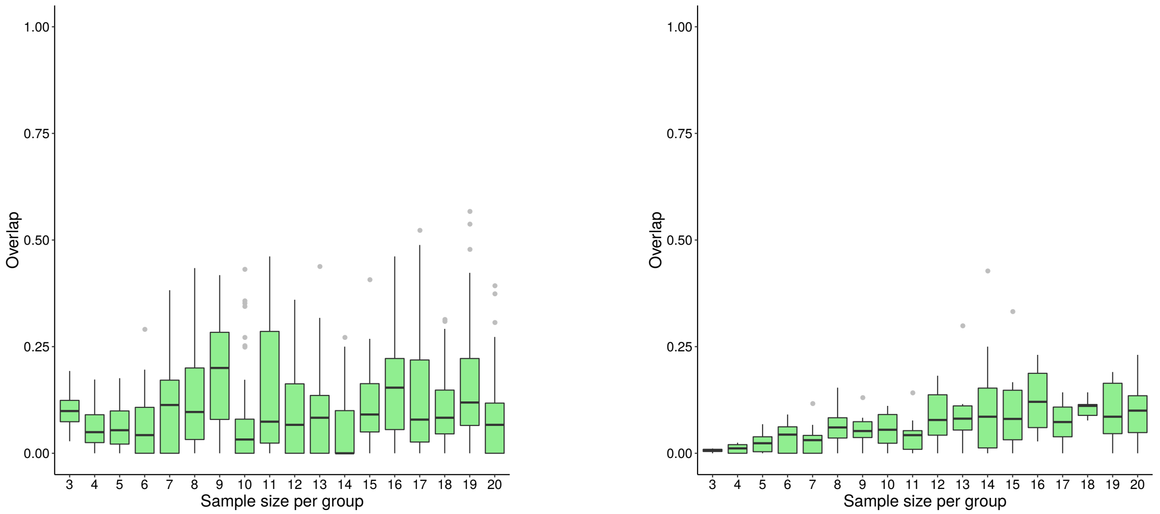


**Figure S17** Box plots showing the distribution of overlap scores resulting from gene set analysis using PADOG when using the original dataset GSE53757 for generating replicate datasets. The panel on the left shows the overlap scores from replicate datasets, while that on the right depicts the overlap scores of each replicate dataset and the whole dataset. See Figure 3 caption for more information.

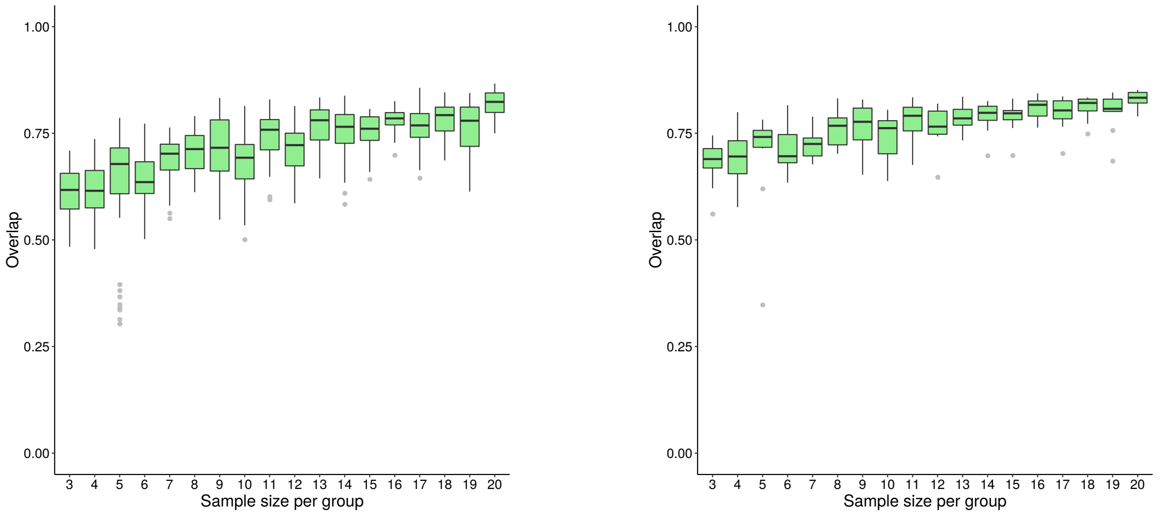


**Figure S18** Box plots showing the distribution of overlap scores resulting from gene set analysis using GSEA-G when using the original dataset GSE53757 for generating replicate datasets. The panel on the left shows the overlap scores from replicate datasets, while that on the right depicts the overlap scores of each replicate dataset and the whole dataset. See Figure 3 caption for more information.

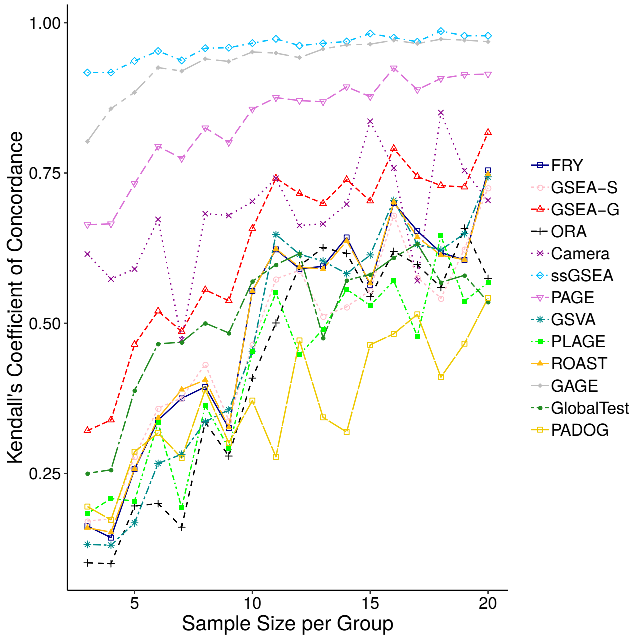


**Figure S19** Kendall's coefficient of concordance for each method under study when using the original dataset GSE10334 for generating replicate datasets. The x-axis shows the sample size. The y-axis shows concordance coefficients of the results of gene set analysis of 10 replicate datasets of the same size.

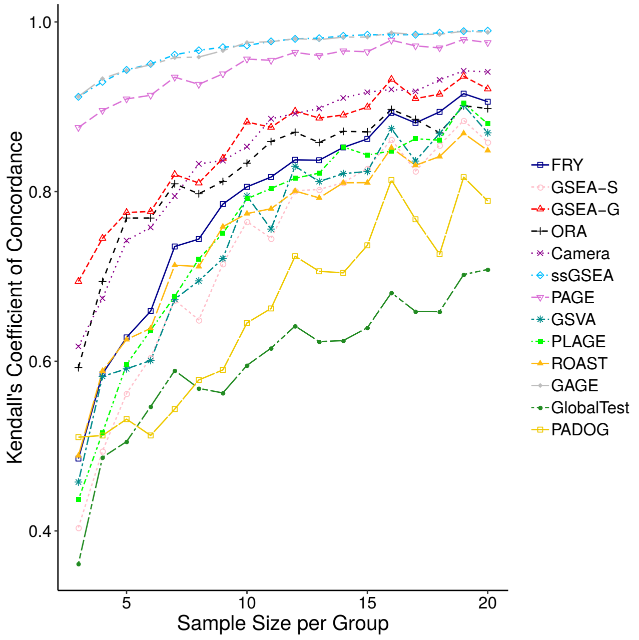


**Figure S20** Kendall's coefficient of concordance for each method under study when using the original dataset GSE13355 for generating replicate datasets. The x-axis shows the sample size. The y-axis shows concordance coefficients of the results of gene set analysis of 10 replicate datasets of the same size.

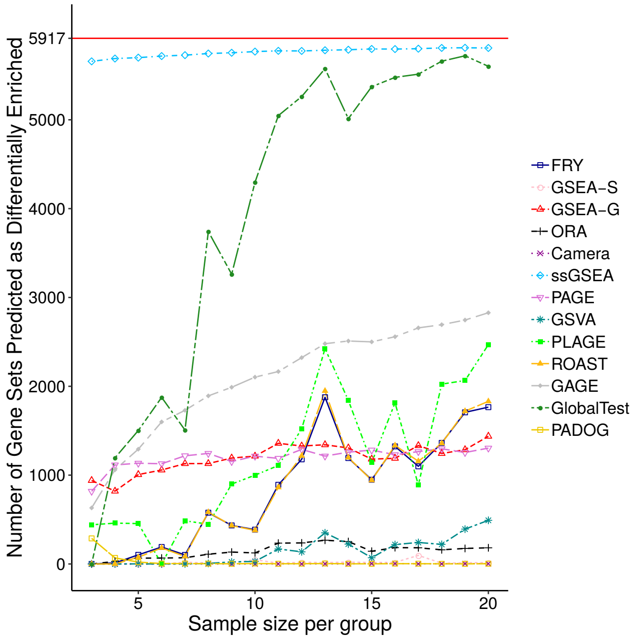


**Figure S21** The number of gene sets predicted as differentially enriched for each method under study when using the original dataset GSE10334 for generating replicate datasets. The x-axis shows the sample size per group. The y-axis shows the average number of gene sets predicted as differentially enriched across 10 replicate datasets of the same size. The red line parallel to the x-axis shows the size of the gene set database being used, i.e. the maximum possible number of gene sets that could be predicted as being differentially enriched.

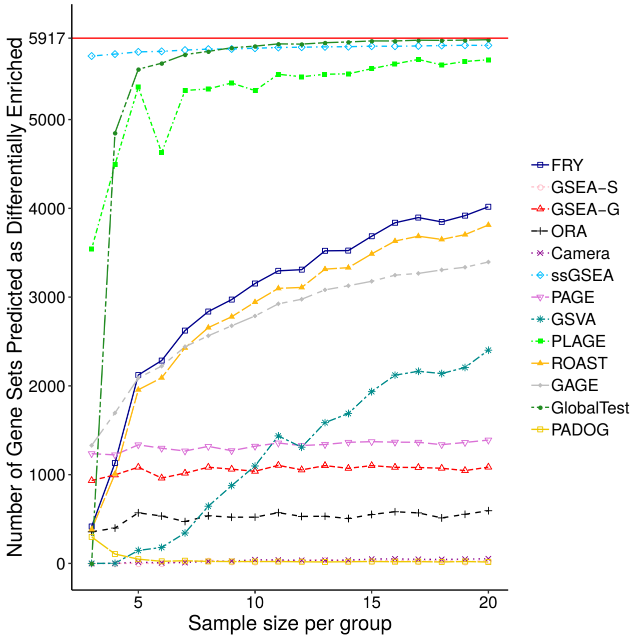


**Figure S22** The number of gene sets predicted as differentially enriched for each method under study when using the original dataset GSE13355 for generating replicate datasets. The x-axis shows the sample size per group. The y-axis shows the average number of gene sets predicted as differentially enriched across 10 replicate datasets of the same size. The red line parallel to the x-axis shows the size of the gene set database being used, i.e. the maximum possible number of gene sets that could be predicted as being differentially enriched.

**Table S2** Average ($\mu$) and standard deviation ($\sigma$) of the number of differentially enriched gene sets reported by each method for control-control experiment when using the original dataset GSE53757 for generating replicate datasets. Since both phenotypes have been randomly chosen from control samples of a real dataset (GSE53757), no differentially enriched gene set is expected. The reported gene sets are considered as false positives. Methods with a large number of reported gene sets suffer from a lack of specificity.

| Sample size per group | | 3 | 4 | 5 | 6 | 7 | 8 | 9 | 10 | 11 | 12 | 13 | 14 | 15 | 16 | 17 | 18 | 19 | 20 |
|---|---|---|---|---|---|---|---|---|---|---|---|---|---|---|---|---|---|---|---|
| FRY | $\mu$ | 0.1 | 0.1 | 0.0 | 0.0 | 0.0 | 0.0 | 0.0 | 0.0 | 0.0 | 0.0 | 0.1 | 0.0 | 0.0 | 0.0 | 0.0 | 0.0 | 0.0 | 0.0 |
| | $\sigma$ | 0.3 | 0.3 | 0.0 | 0.0 | 0.0 | 0.0 | 0.0 | 0.0 | 0.0 | 0.0 | 0.3 | 0.0 | 0.0 | 0.0 | 0.0 | 0.0 | 0.0 | 0.0 |
| GSEA-S | $\mu$ | 0.0 | 0.0 | 0.0 | 0.0 | 17.2 | 6.5 | 10.2 | 13.1 | 16.1 | 7.0 | 14.0 | 10.4 | 16.4 | 11.1 | 9.5 | 10.3 | 11.3 | 9.9 |
| | $\sigma$ | 0.0 | 0.0 | 0.0 | 0.0 | 18.5 | 4.2 | 8.8 | 12.8 | 14.9 | 4.4 | 16.8 | 9.6 | 15.9 | 6.3 | 7.8 | 7.4 | 7.4 | 11.9 |
| GSEA-G | $\mu$ | 621.8 | 578.1 | 596.6 | 555.8 | 609.1 | 559.5 | 677.8 | 792.0 | 884.5 | 789.0 | 720.6 | 511.8 | 639.9 | 556.5 | 565.6 | 758.0 | 707.5 | 425.5 |
| | $\sigma$ | 463.4 | 580.2 | 454.5 | 450.4 | 441.4 | 476.2 | 420.7 | 472.5 | 526.7 | 528.7 | 442.4 | 348.0 | 531.0 | 471.6 | 441.3 | 655.6 | 326.1 | 338.3 |
| ORA | $\mu$ | 0.0 | 0.0 | 0.0 | 0.0 | 0.0 | 0.0 | 0.0 | 0.0 | 0.0 | 0.0 | 0.0 | 0.0 | 0.0 | 0.0 | 0.0 | 0.0 | 0.0 | 0.0 |
| | $\sigma$ | 0.0 | 0.0 | 0.0 | 0.0 | 0.0 | 0.0 | 0.0 | 0.0 | 0.0 | 0.0 | 0.0 | 0.0 | 0.0 | 0.0 | 0.0 | 0.0 | 0.0 | 0.0 |
| Camera | $\mu$ | 0.0 | 0.0 | 0.8 | 0.2 | 0.0 | 0.3 | 38.2 | 28.3 | 54.1 | 18.5 | 67.0 | 38.8 | 102.2 | 55.5 | 62.5 | 72.9 | 55.3 | 129.3 |
| | $\sigma$ | 0.0 | 0.0 | 2.4 | 0.4 | 0.0 | 0.5 | 107.6 | 59.9 | 83.2 | 18.5 | 80.7 | 29.9 | 98.2 | 64.6 | 61.4 | 55.1 | 34.8 | 78.4 |
| ssGSEA | $\mu$ | 5157.7 | 5174.8 | 5185.0 | 5220.1 | 5219.1 | 5231.9 | 5233.5 | 5249.3 | 5249.2 | 5256.5 | 5251.9 | 5261.5 | 5264.1 | 5271.0 | 5278.2 | 5276.9 | 5282.5 | 5283.0 |
| | $\sigma$ | 1712.0 | 1717.0 | 1720.3 | 1731.6 | 1731.1 | 1735.4 | 1735.9 | 1741.1 | 1741.0 | 1743.4 | 1741.9 | 1745.0 | 1745.9 | 1748.3 | 1750.6 | 1750.2 | 1752.1 | 1752.2 |
| PAGE | $\mu$ | 1189.5 | 1071.5 | 1082.9 | 1095.2 | 1155.4 | 1206.6 | 1246.0 | 1343.0 | 1169.7 | 1225.2 | 1221.6 | 1020.3 | 1228.2 | 1139.4 | 1115.6 | 1128.4 | 1208.5 | 1055.5 |
| | $\sigma$ | 426.8 | 509.0 | 472.1 | 424.1 | 417.4 | 456.1 | 473.4 | 477.4 | 530.3 | 516.4 | 467.7 | 409.7 | 538.5 | 412.9 | 546.4 | 472.9 | 471.7 | 473.0 |
| GSVA | $\mu$ | 0.0 | 0.0 | 0.0 | 0.0 | 0.0 | 0.0 | 0.0 | 0.0 | 0.0 | 0.0 | 0.0 | 0.0 | 0.0 | 0.0 | 0.0 | 0.0 | 0.0 | 0.0 |
| | $\sigma$ | 0.0 | 0.0 | 0.0 | 0.0 | 0.0 | 0.0 | 0.0 | 0.0 | 0.0 | 0.0 | 0.0 | 0.0 | 0.0 | 0.0 | 0.0 | 0.0 | 0.0 | 0.0 |
| PLAGE | $\mu$ | 0.0 | 0.0 | 0.0 | 0.0 | 0.0 | 0.0 | 0.0 | 0.0 | 0.0 | 0.0 | 0.0 | 0.0 | 0.0 | 0.0 | 0.0 | 0.0 | 0.0 | 0.0 |
| | $\sigma$ | 0.0 | 0.0 | 0.0 | 0.0 | 0.0 | 0.0 | 0.0 | 0.0 | 0.0 | 0.0 | 0.0 | 0.0 | 0.0 | 0.0 | 0.0 | 0.0 | 0.0 | 0.0 |
| ROAST | $\mu$ | 0.0 | 0.0 | 0.0 | 0.0 | 0.0 | 0.0 | 0.0 | 0.0 | 0.0 | 0.0 | 0.0 | 0.0 | 0.0 | 0.0 | 0.0 | 0.0 | 0.0 | 0.0 |
| | $\sigma$ | 0.0 | 0.0 | 0.0 | 0.0 | 0.0 | 0.0 | 0.0 | 0.0 | 0.0 | 0.0 | 0.0 | 0.0 | 0.0 | 0.0 | 0.0 | 0.0 | 0.0 | 0.0 |
| GAGE | $\mu$ | 941.9 | 1291.3 | 1472.2 | 1718.0 | 1979.4 | 2073.3 | 2214.6 | 2317.6 | 2373.8 | 2452.0 | 2540.7 | 2553.8 | 2632.5 | 2647.8 | 2683.5 | 2741.0 | 2746.3 | 2779.1 |
| | $\sigma$ | 364.0 | 488.9 | 510.9 | 592.4 | 665.2 | 698.2 | 740.2 | 772.1 | 792.0 | 824.3 | 847.1 | 848.3 | 874.6 | 880.6 | 892.6 | 910.4 | 911.9 | 923.5 |
| GlobalTest | $\mu$ | 0.0 | 0.0 | 0.0 | 0.0 | 0.0 | 0.0 | 0.0 | 0.0 | 0.0 | 0.0 | 0.0 | 0.0 | 0.0 | 0.0 | 0.0 | 0.0 | 0.0 | 0.0 |
| | $\sigma$ | 0.0 | 0.0 | 0.0 | 0.0 | 0.0 | 0.0 | 0.0 | 0.0 | 0.0 | 0.0 | 0.0 | 0.0 | 0.0 | 0.0 | 0.0 | 0.0 | 0.0 | 0.0 |
| PADOG | $\mu$ | 264.8 | 78.6 | 24.7 | 6.3 | 7.3 | 6.2 | 7.1 | 5.0 | 4.1 | 4.4 | 6.5 | 6.3 | 6.6 | 7.6 | 4.2 | 3.8 | 6.7 | 5.7 |
| | $\sigma$ | 106.6 | 37.5 | 12.1 | 4.9 | 4.5 | 6.0 | 7.7 | 3.3 | 4.9 | 4.3 | 5.6 | 6.6 | 5.3 | 5.7 | 4.5 | 5.2 | 4.6 | 4.8 |
